# In-silico human electro-mechanical ventricular modelling and simulation for drug-induced pro-arrhythmia and inotropic risk assessment

**DOI:** 10.1016/j.pbiomolbio.2020.06.007

**Published:** 2021-01

**Authors:** Francesca Margara, Zhinuo J. Wang, Francesc Levrero-Florencio, Alfonso Santiago, Mariano Vázquez, Alfonso Bueno-Orovio, Blanca Rodriguez

**Affiliations:** a Department of Computer Science, University of Oxford, Parks Road OX1 3QD, Oxford, United Kingdom; b Barcelona Supercomputing Center - Centro Nacional de Supercomputación, C/Jordi Girona 29, Barcelona, 08034, Spain; c ELEM Biotech, Spain

**Keywords:** Human ventricular action potential, Human cardiac contraction, In-silico drug trials, Drug safety, Computer simulation, Computational modelling, AP, action potential, APD, action potential duration, CaT, calcium transient, EAD, early afterdepolarisation

## Abstract

Human-based computational modelling and simulation are powerful tools to accelerate the mechanistic understanding of cardiac patho-physiology, and to develop and evaluate therapeutic interventions. The aim of this study is to calibrate and evaluate human ventricular electro-mechanical models for investigations on the effect of the electro-mechanical coupling and pharmacological action on human ventricular electrophysiology, calcium dynamics, and active contraction.

The most recent models of human ventricular electrophysiology, excitation-contraction coupling, and active contraction were integrated, and the coupled models were calibrated using human experimental data. Simulations were then conducted using the coupled models to quantify the effects of electro-mechanical coupling and drug exposure on electrophysiology and force generation in virtual human ventricular cardiomyocytes and tissue. The resulting calibrated human electro-mechanical models yielded active tension, action potential, and calcium transient metrics that are in agreement with experiments for endocardial, epicardial, and mid-myocardial human samples. Simulation results correctly predicted the inotropic response of different multichannel action reference compounds and demonstrated that the electro-mechanical coupling improves the robustness of repolarisation under drug exposure compared to electrophysiology-only models. They also generated additional evidence to explain the partial mismatch between in-silico and in-vitro experiments on drug-induced electrophysiology changes.

The human calibrated and evaluated modelling and simulation framework constructed in this study opens new avenues for future investigations into the complex interplay between the electrical and mechanical cardiac substrates, its modulation by pharmacological action, and its translation to tissue and organ models of cardiac patho-physiology.

## 1 Introduction

Human-based computational approaches have now built momentum beyond academia, reaching clinical, industrial, and regulatory environments. They provide virtual tools, embedding our knowledge on human physiology, to investigate disease, and develop and evaluate therapeutic interventions (Rodriguez et al., 2016; Morrison et al., 2018; Viceconti et al., 2020). Recent studies have, for example, demonstrated the ability of biophysical human-based modelling and simulation for the assessment of the safety and efficacy of pharmacological therapies (Sarkar and Sobie, 2011; Passini et al., 2017, 2019; Li et al., 2019).

Over the last six decades, computational modelling and simulation have played a key role in cardiac science. They have accelerated the mechanistic understanding of cardiac patho-physiology, usually through separate paths for electrophysiology and contractility. Iterations between experimental and modelling and simulation science have enabled the development and validation of biophysical models (Rudy and Silva, 2006; Noble, 2011; Carusi et al., 2012). Technical advancement and availability of human tissue have fostered the collection of human-specific datasets, characterising different aspects of ventricular electro-mechanics. These include excitation-contraction coupling and calcium dynamics (Lou et al., 2011; O’Hara et al., 2011; Coppini et al., 2013), as well as contractile (Mulieri et al., 1992; Pieske et al., 1996; Rossman, 2004; Land et al., 2017; Coppini et al., 2019) and passive material properties (Demer and Yin, 1983; Yin et al., 1987; Holzapfel and Ogden, 2009). Experimental data at different scales from subcellular to whole-organ dynamics have then been integrated into multiscale computer models of human physiology (Augustin et al., 2016; Land et al., 2017; Prakosa et al., 2018; Levrero-Florencio et al., 2020).

Building on previous models (ten Tusscher and Panfilov, 2006; Grandi et al., 2010; Carro et al., 2011; O’Hara et al., 2011), Tomek et al. (2019) recently proposed the human ventricular ToR-ORd model of cellular electrophysiology and excitation-contraction coupling. The ToR-ORd model was developed based on the overall structure of the ORd model (O’Hara et al., 2011), but with key improvements in the L-type calcium, potassium and chloride currents to improve the agreement with experimental data in terms of action potential (AP) morphology and response to drug block. The development of the ToR-ORd model also involved the use of independent experimental datasets for calibration and validation.

Moreover, Land et al. (2017) recently proposed a new model of human cardiac contraction, building on previous theoretical research (Niederer et al., 2006; Rice et al., 2008; Land et al., 2012) and novel measurements of tension development in human cardiomyocytes.

The aim of this study is to investigate the effects of electro-mechanical coupling in human ventricular electrophysiology, calcium dynamics and active contraction, and their modulation by pharmacological action using multiscale modelling and simulation. To address this goal, the most recent models of human ventricular electrophysiology and active contraction are coupled and calibrated using experimental data. For electrophysiology, we consider both the ToR-ORd and its predecessor, the ORd model, and we hypothesise that the improvements in the excitation-contraction coupling in the ToR-ORd (with respect to the ORd model) will yield a better agreement between simulations and experimental data.

Simulations are conducted to quantify the effect of electro-mechanical coupling on the action potential, calcium dynamics, and active contraction in healthy virtual human ventricular cardiomyocytes and tissue and under drug exposure. This study provides a human-based integrated pipeline for the simultaneous simulation of electrophysiology, calcium dynamics and contractility in ventricular cardiomyocytes. The proposed modelling and simulation framework offers the opportunity to extend human-based investigations into the mechanisms of disease and drug action to incorporate heterogeneous electrical and mechanical features of cardiac function and their strong coupling.

## 2 Material and methods

### 2.1 Experimental data

Table 1 summarises the experimental recordings used in this study to calibrate and evaluate the human electro-mechanical models. Active tension data (Mulieri et al., 1992; Pieske et al., 1996; Rossman, 2004) obtained from isolated human preparations at body temperature were considered for the model calibration. A heterogeneous and comprehensive set of data was used for model validation, including human recordings of action potential (Britton et al., 2017b) and calcium transients (Coppini et al., 2013), electrophysiology (Lou et al., 2011) and contractile (Haynes et al., 2014) transmural heterogeneity data, and drug responses (Page et al., 2016; Nguyen et al., 2017). Moreover, force (Mulieri et al., 1992), action potential (O’Hara et al., 2011) and calcium transient (Schmidt et al., 1998) frequency dependence, and force-length dependence (Vahl et al., 1997; Holubarsch, 1998) data were considered.

**Table 1:** **Description of the human experimental data used for model calibration and evaluation.** tp: time to peak, rt50/rt90/rt95: time to 50/90/95% decay, APD50/APD90: action potential (AP) duration at 50/90% repolarisation. Calibration data are reported as Mean ± SEM. AP data are reported as [minimum maximum]. CaT data are reported as Median±STD.

	Reference	Tissue preparation	Data type	Biomarkers
**Calibration**	Pieske et al. (1996)	Left ventricular trabeculae from non-failing hearts (paced at 1 Hz, 37 °C)	Active Tension (muscles were gradually stretched along their length-tension curve in 0.05 mm steps until maximum isometric tension was reached)	amplitude (mN/mm^2^): 13.7 ± 1.8 tp (ms): 165 ± 7 rt50 (ms): 116 ± 6 rt95 (ms): 334 ± 43
	Mulieri et al. (1992)	Biopsy strips of left ventricular myocardium of hearts from coronary artery bypass surgery (paced at 1.2 Hz, 37 °C)	Active Tension (muscles were gradually stretched in 0.05 mm increments to the length at which active twitch tension was maximum)	peak (mN/mm^2^): 22.8 ± 1.4 tp (ms): 157 ± 10 rt50 (ms): 117 ± 8 rt95 (ms): 477 ± 31 (time to complete relaxation)
	Rossman et al. (2004)	Trabeculae from the right ventricular free wall of donor heart (paced at 0.5 or 2.5 Hz, 37 °C)	Active Tension (the final length was set at 80% of the difference between *L*max and *L*0, where an active developed force can first be identified)	developed force (mN/mm^2^): 16.7 ± 1.6 (0.5 Hz), 30.3 ± 4.6 (2 Hz) tp (ms): 235 ± 13.4 (0.5 Hz), 151 ± 6.1 (2 Hz) rt50 (ms): 153 ± 71 (0.5 Hz), 98 ± 7.7 (2 Hz) rt95 (ms): 309 ± 13.7 (0.5 Hz), 173 ± 10.7 (2 Hz)
**Evaluation**	Britton et al. (2017b)	Right ventricular trabeculae and papillary tissue preparations from non-failing human hearts, perfused and paced at 1 Hz	Action Potential	tp (ms): [3.1 14.0] APD50 (ms): [106.6 349.4] APD90 (ms): [178.1 442.7]
	Coppini et al. (2013)	Viable single myocytes from septal specimens of control patients	Calcium Transient	tp (ms): 47.8 ± 10 rt50 (ms): 151.1 ± 89.2 rt90 (ms): 315.6 ± 161.2
	Lou et al. (2011)	Isolated left ventricular wedge preparations from non-failing human hearts	Electrophysiological Heterogeneity	AP repolarisation sequence: mid>endo>epi CaT repolarisation sequence: mid>endo>epi
	Haynes et al. (2014)	Permeabilised sub-epicardial, mid-myocardial, and sub-endocardial specimens from left ventricular free wall of non-failing human hearts	Contractile Heterogeneity	Isometric tension distribution: higher at mid-myocardium
	Nguyen et al. (2017)	Adult human primary ventricular myocytes isolated from donor hearts	Active Tension	Tension reduction under drug exposure
	Guo et al. (2011)	Isolated non-failing human ventricular endocardial myocytes	Action Potential	EADs formation under drug exposure
	Page et al. (2016)	Human ventricular trabeculae from ethically consented organ donors	Action Potential	APD prolongation and EADs formation under drug exposure
	O’Hara et al. (2011)	Nonfailing human hearts	Action Potential	AP-frequency dependence
	Schmidt et al. (1998)	Nonfailing human hearts	Intracellular Calcium Peak and Decay	CaT-frequency dependence
	Mulieri et al. (1992)	Biopsy strips of left ventricular myocardium of hearts from coronary artery bypass surgery (paced at 1.2 Hz, 37 °C)	Active Tension (muscles were gradually stretched in 0.05-mm increments to the length at which active twitch tension was maximum)	Force-frequency dependence
	Vahl et al. (1997)	Isolated intact human left ventricular myocardium from 8 normal donor hearts (paced at 1 Hz, 37 °C)	Developed Force (measured for L/Lmax between 0.8 and 1)	Length-dependence of developed force
	Holubarsch et al. (1998)	Human left ventricular non-failing preparations from 8 donor hearts (paced at 0.5 Hz, 37 °C).	Developed Force	Length-dependence of developed force

### 2.2 Description of the electro-mechanical coupling of human-based models

Previously-published human electrophysiology, calcium dynamics and active tension models were coupled through forward (excitation-contraction coupling) and reverse (mechano-electric feedback) processes. Specifically, the new ToR-ORd model published in Tomek et al. (2019) and the modified version of the ORd model (O’Hara et al., 2011) published in Dutta et al. (2017) were considered for electrophysiology and excitation-contraction coupling. Both of which included formulations for the following currents: the sodium current (INa), the transient outward potassium current (Ito), the L-type calcium current (ICaL), the rapid delayed rectifier potassium current (IKr), the slow delayed rectifier potassium current (IKs), the inward rectifier potassium current (IK1), the sodium-calcium exchange current (INaCa), the sodium-potassium ATPase current (INaK), the background currents, the sarcolemmal calcium pump current (IpCa). The calcium-related model components in both the ToR-ORd and ORd also describe calcium release from the ryanodine receptors, calcium uptake through SERCA pumps, calcium translocation between the two sarcoplasmic reticulum (SR) compartments, the network and junctional compartments, calcium buffers (calmodulin, troponin, anionic SR and sarcolemmal binding sites, calsequestrin), and calcium/calmodulin dependant protein kinase.

The key improvements of the ToR-ORd compared to the ORd models are an in-depth re-evaluation of the ICaL formulation, the re-assessment of the IKr, the replacement of the INa and IK1, as well as the incorporation of additional currents (calcium-sensitive chloride and background chloride currents). This allowed an improved representation of the AP plateau, action potential duration (APD) adaptation, and responses to sodium current block (Tomek et al., 2019).

The Land model (Land et al., 2017) was adopted for active tension generation. It is based on novel measurements obtained from human cardiomyocytes at body temperature and it features troponin C and tropomyosin kinetics and a three-state crossbridge model that also accounts for crossbridge distortion.

The bidirectional electro-mechanical coupling was achieved as previously reported in Timmermann et al. (2017) and Levrero-Florencio et al. (2020). Briefly, the free intracellular calcium concentration computed in the electrophysiology models (ToR-ORd and ORd) served as the input for the Land model, and the amount of calcium bound to troponin C computed in the Land model was fed back into the ToR-ORd and ORd models and used to update the free intracellular calcium concentration. See the supplementary material (S1) for further details of the coupling implementation.

**Fig. 1:**
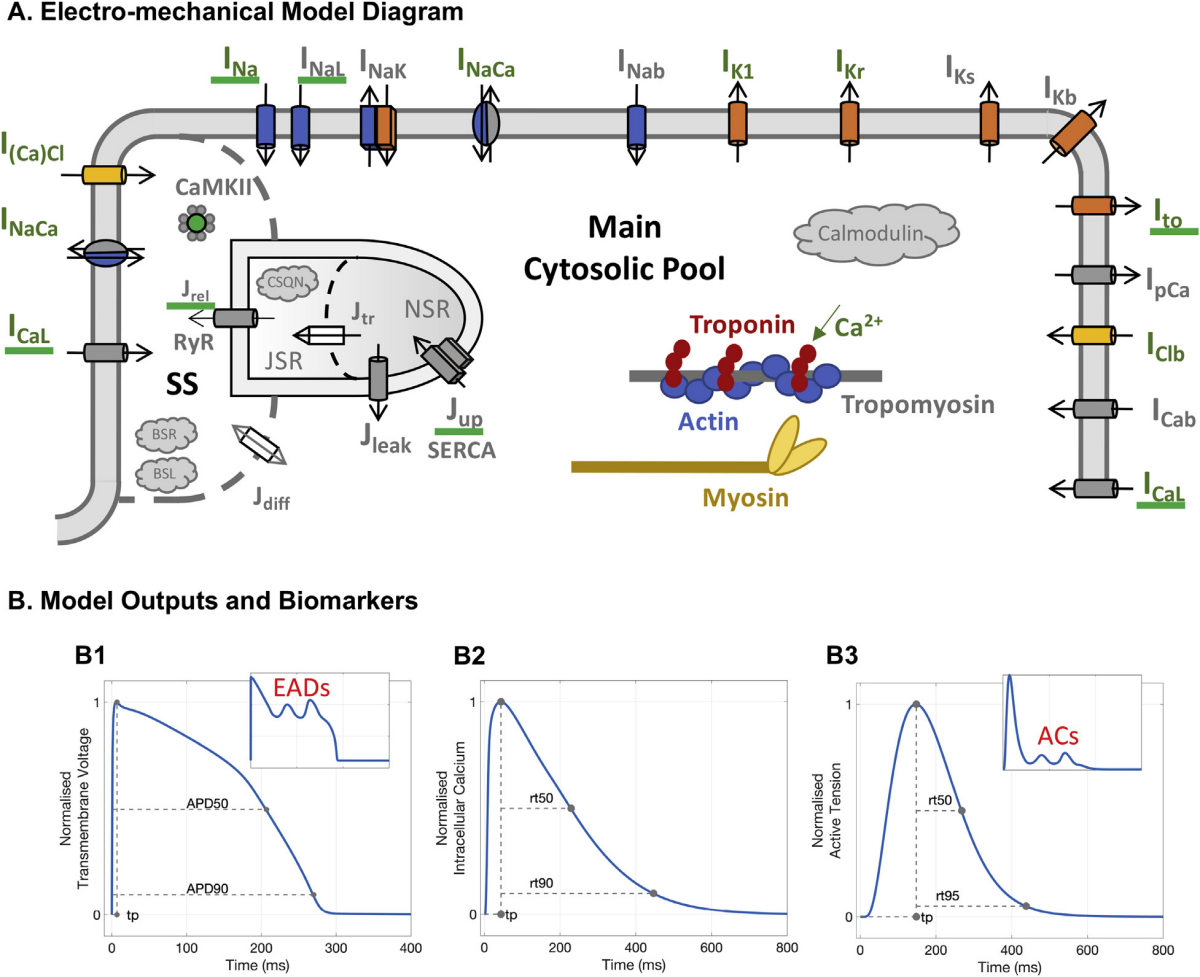
**Human-based biophysical models and biomarkers of electro-mechanical function.** A: Schematic representation of ionic currents, calcium dynamics and contractile properties considered. Here the electrophysiology model diagram represents the ToR-ORd model (adapted from Tomek et al., 2019 as allowed by the CC-BY license). B: Biomarkers computed. B1: Typical AP waveform and relative biomarkers (tp: time to AP peak; APD50/APD90: AP duration at 50/90% repolarisation). Inset: AP repolarisation abnormalities (early afterdepolarisations, EADs). B2: Typical CaT waveform and relative biomarkers (tp: time to CaT peak; rt50/rt90: time to 50/90% decay from CaT peak). B3: Typical active tension waveform and relative biomarkers (tp: time to active tension peak; rt50/rt95: time to 50/95% decay from active tension peak). Inset: Active tension abnormalities (aftercontractions, ACs).

Fig. 1 presents an illustration of the coupling between the ToR-ORd and Land models and the biomarkers considered in this study. It also illustrates the abnormalities in AP and active tension (early afterdepolarisations, EADs, and aftercontractions, respectively) that may arise under drug exposure. Specifically, the biomarkers computed after each electro-mechanical simulation were: time-to-peak (tp) response of AP, calcium transient (CaT), and active tension; APD at 50% and 90% of repolarisation (APD50 and APD90, respectively); and relaxation times of CaT and active tension at 50% and 90/95% decay (rt50 and rt90/rt95, respectively). Occurrences of EADs and aftercontractions were identified as a positive change in the slope of the AP repolarisation phase and of the contractility relaxation transient, before the following stimulus-induced AP and contraction, respectively (Nguyen et al., 2017). Occurrences of contractility escapes were identified as electrical stimuli that did not result in a contraction transient.

### 2.3 Recalibration of the active tension model

**Fig. 2:**
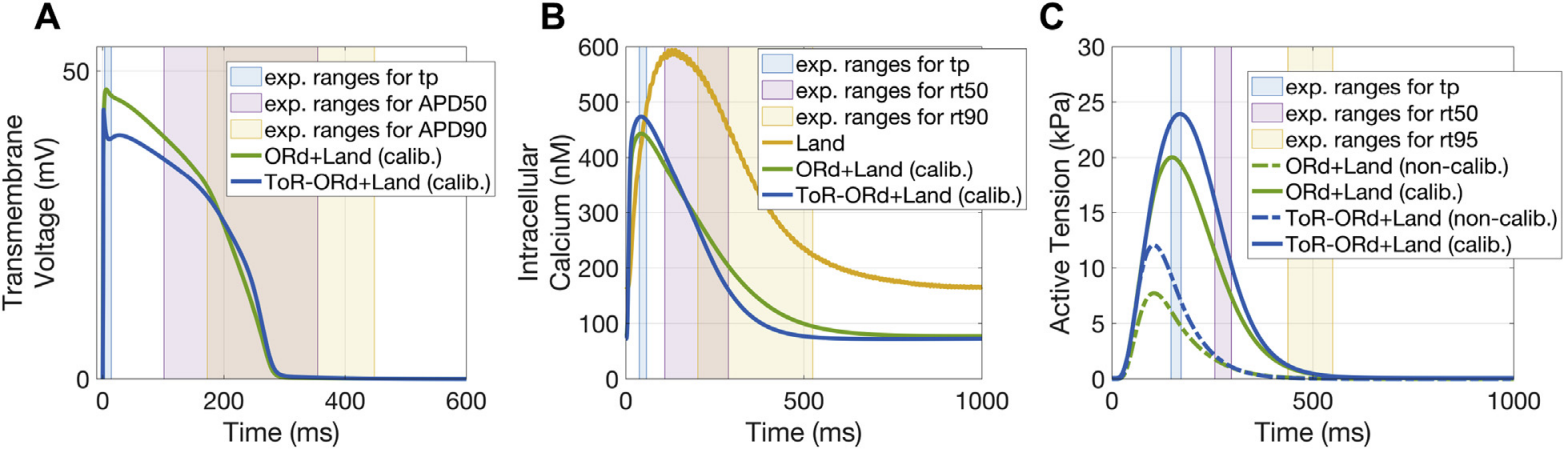
**Human electro-mechanical model calibration and comparison with experimental data.** Comparisons between the AP (A), CaT (B), and active tension (C) of the calibrated models and human experimental data (A: Britton et al., 2017b; B: Coppini et al., 2013; C: Mulieri et al., 1992; Pieske et al., 1996; Rossman et al., 2004). ToR-ORd+Land model in blue and ORd+Land model in green. Calibrated models have AP, CaT, and active tension biomarkers that are within experimental ranges. Panel B also shows the CaT used to drive contraction in the original Land model (yellow). Calibration does not affect the AP or CaT (A-B). Panel C shows how the simulated active tensions better replicate experimental data after calibration.

The coupled electro-mechanical models (from now on referred to as ToR-ORd+Land and ORd+Land) were calibrated against human experimental recordings to achieve physiological active tension. This was necessary because the CaT from the ToR-ORd and ORd models was in close agreement with human recordings (Coppini et al., 2013) but was different from the transient used by Land to drive contraction (Fig. 2-B). Therefore, the Land model parameters had to be re-parameterised to achieve physiological active tension, when driven by the physiological CaTs from the ToR-ORd (and ORd) model. The calibrated Land model parameters were the Hill coefficient of cooperative activation and the tropomyosin rate constant (Table S1).

As in Land, isometric twitch data in intact human preparations from the literature (Table 1) were used to extrapolate target active tension biomarkers. Details on the fitting procedure, including the choice of model parameters to be varied, the biomarkers’ ranges, the cost function and the algorithm used can be found in the supplementary material (S2). The calibrated ToR-ORd+Land and ORd+Land models were then used to evaluate the various physiological effects of electro-mechanical coupling.

### 2.4 Evaluation of the human electro-mechanical models and transmural heterogeneity

Firstly, we compared the simulated APs and CaTs using the ToR-ORd+Land and ORd+Land models with human experimental data (Coppini et al., 2013; Britton et al., 2017b), as in Tomek et al. (2019), to evaluate the electrophysiological consequences of electro-mechanical coupling. We then incorporated transmural heterogeneities in ionic properties to generate human mid-myocardial and epicardial cell models from the endocardial version described above. Details of the heterogeneity implementation can be found in the supplementary material (S3).

### 2.5 Simulation of drug-induced effects on human electro-mechanical function

We simulated the drug-induced effects on cellular and tissue electro-mechanical function and compared the results with reported effects on human preparations. We focused on the following multichannel action reference compounds: Dofetilide (antiarrhythmic drug class III), Verapamil (antiarrhythmic drug class IV) and Quinidine (antiarrhythmic drug class Ia). Compounds selection was based on preclinical (Kramer et al., 2013; Crumb et al., 2016; Nguyen et al., 2017) and clinical (Ritchie et al., 2006; Johannesen et al., 2014; Vicente et al., 2016) data availability for electrophysiology and contractility in human subjects. The selected drugs were reference compounds with well-characterised clinical outcomes and different pro-arrhythmic profiles: Verapamil is considered safe with no Torsade de Pointes (TdP) risk, while both Dofetilide and Quinidine prolong the QT interval and are clearly associated with TdP risk (CredibleMeds®, Woosley and Romer, 1999).

Drug effects were incorporated in the electro-mechanical models using IC50 and Hill coefficient data (Passini et al., 2017), by means of simple pore-block models of drug action. The full list of compounds, IC50/Hill coefficients and the maximal effective free therapeutic concentrations used to simulate drug action can be found in the supplementary material (S4). To estimate inotropic effects, the percentage of tension reduction relative to control was computed and summarised as Hill curves. Simulated active tension was compared to experimentally recorded sarcomere shortening in human cardiomyocytes (Nguyen et al., 2017) as a qualitative measurement of drug-induced changes in cardiac inotropy. Drug-induced effects on AP were compared against AP recordings obtained in human trabeculae (Page et al., 2016).

### 2.6 Single cell implementation and stimulation protocols

Cellular simulations were conducted using MatLab (Mathworks Inc. Natwick, MA, USA) using the ordinary differential equation solver *ode15s*. Stimulus currents of −53 μA/μF with 1 ms duration, and of −80 μA/μF with 0.5 ms duration were used, as in the original ToR-ORd and ORd models, respectively. The extension ratio (λ, sarcomere length over sarcomere length at rest) was set to 1, unless otherwise stated. For each baseline simulation, steady-state was reached at 1 Hz pacing before biomarkers were computed. For the in-silico drug trials, after reaching steady-state at 1 Hz pacing frequency, drugs were applied, and the simulations ran for an additional 200 beats at 1 Hz pacing frequency (unless otherwise stated). The final beats were inspected for depolarisation/repolarisation abnormalities, aftercontractions, and contractility escapes.

### 2.7 Fully-coupled electro-mechanical simulations in 3D tissue slabs

To analyse the functional consequences of drug exposure on tissue-level cardiac electro-mechanics, we incorporated the biophysically-detailed model of cellular electro-mechanical coupling into the 3D framework of electrical propagation and tissue deformation recently described in Levrero-Florencio et al. (2020), solved using the multi-physics computational code *Alya* (Santiago et al., 2018). This includes coupled anisotropic descriptions of the mechanical behaviour of ventricular muscle (Holzapfel and Ogden, 2009) and cardiac electrical propagation (monodomain formulation, Leon and Horácek (1991)).

Cuboid geometries were used to model small tissue preparations that are often exploited in experimental investigations of cardiac contraction (Mulieri et al., 1992; Pieske et al., 1996; Rossman, 2004) and preclinical drug testing (Page et al., 2016). They offer the advantage of up-scaling investigations from cellular to 3D preparations, incorporating cell coupling effects, without the computational complexity that arises from more complex geometries.

Simulations were performed on a 1 cm cube made of endocardial cells with constant fibre directions along the z axis. Simulated tissue samples are electrically and mechanically transversely isotropic. Dirichlet boundary conditions were applied at the bottom face (z = 0), fixing it in all directions. A stimulus current of −100 μA/μF for 1 ms was applied to one of the fixed corners at 1 Hz pacing until steady-state was reached. Drug-induced effects were quantified through the computation of the longitudinal shortening together with the analysis of abnormalities in electrical conduction and mechanical contraction.

## 3 Results

### 3.1 Evaluation of the human electro-mechanical cellular models

Fig. 2 illustrates the AP, CaT, and active tension time course for the novel ToR-ORd+Land and ORd+Land models, compared to the Land model. It shows the need for recalibration of the mechanical Land model in order to obtain physiological active tensions with the coupled models. In particular, Fig. 2-B shows the large amplitude and the delayed time-to-peak of the CaT used in the original Land model, compared to the new coupled models. Due to such differences, the active tensions simulated by the non-calibrated models are significantly lower (Fig. 2-C, dashed lines) than reported physiological values. This follows the well-known non-linear relationship between calcium and force. Such inconsistencies are corrected by the calibration process (Fig. 2-C, solid lines), which yields simulated active tensions for both models in line with the experimental data considered in terms of amplitude, peak development and relaxation kinetics. This procedure does not affect the morphology nor the kinetics of AP (Fig. 2-A) and CaT (Fig. 2-B), retaining good agreement with human experimental data (Coppini et al., 2013; Britton et al., 2017b).

Further details on how the calibration affects the contractility model and the steady-state calcium-force relationship can be found in the supplementary material (S5). The supplementary material (S6) also includes information on the preserved force-frequency and length-dependence properties of the calibrated ToR-ORd+Land and ORd+Land models.

**Fig. 3:**
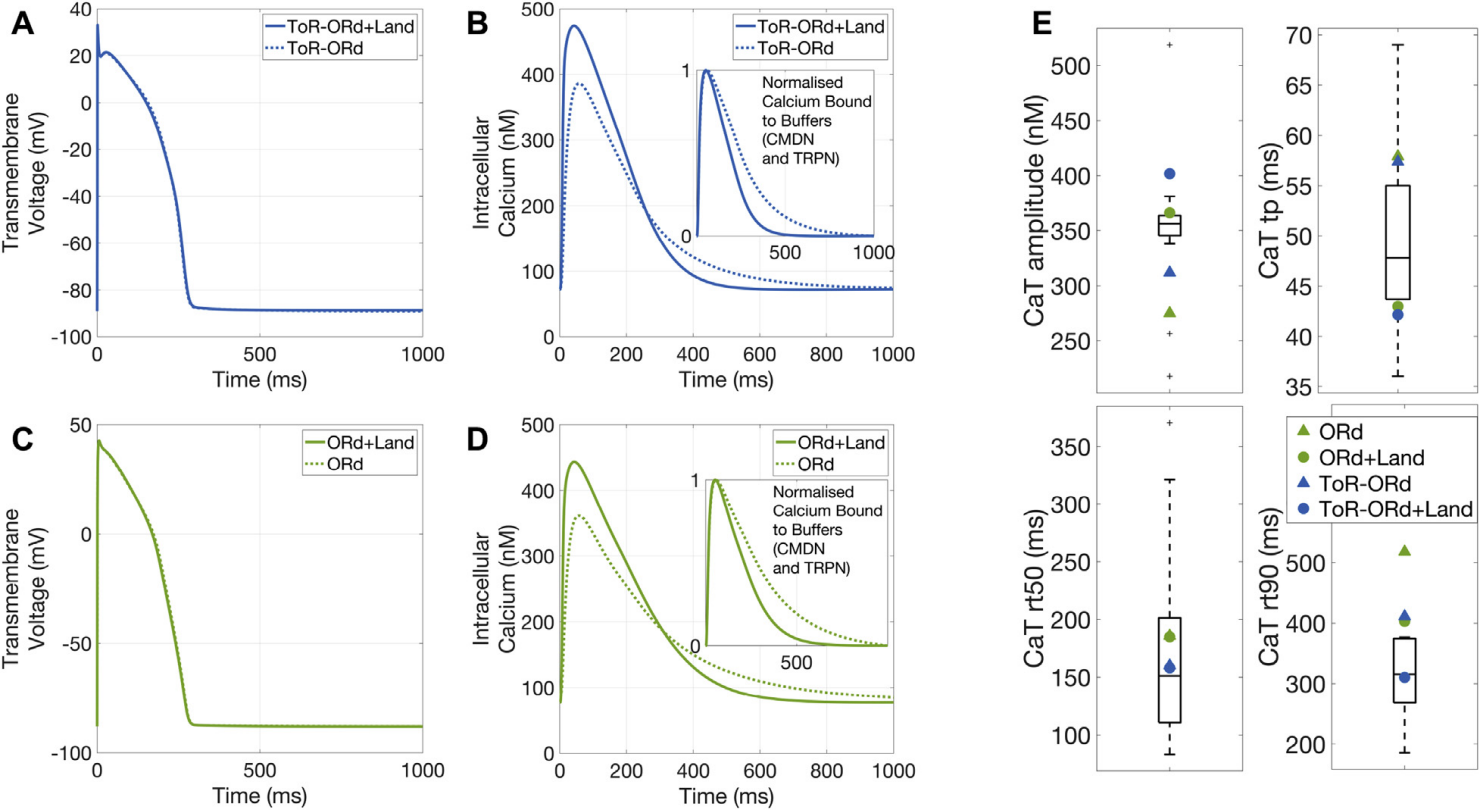
**Electrophysiological consequences of dynamic calcium binding to troponin C.** A/C: Electro-mechanical coupling does not affect AP waveforms. B/D: The dynamic calcium binding to troponin C formulation changes the time course of CaTs and predicts less calcium binding to troponin C compared with the previous steady-state approximation (insets). E: Quantification of changes in CaT biomarkers induced by the dynamic calcium binding to troponin C. Black box plots represent experimental data.

We next analysed how the coupling with the contractility model affects cellular electrophysiology. The AP morphology and kinetics of the electro-mechanically coupled models perfectly resemble those of the purely electrophysiological models (Fig. 3-A, C). However, the CaT exhibits larger amplitudes and faster kinetics (Fig. 3-B, D). Fig. 3-E displays the calcium transient biomarkers as obtained in experiments and simulation. The black box plots represent the experimental data (Coppini et al., 2013) obtained in non-failing non-hypertrophic control cells through fluorescence imaging. The simulation results are obtained with the electro-mechanical models (circles) and their corresponding electrophysiology-only models (triangles). The electro-mechanical coupling brings an improvement in better capturing the experimental data, especially for the calcium duration at 90% decay. The simulated biomarker values fall within the experimental range, even though in some cases outside of the 25–75 percentiles range.

The differences between the electrophysiology-only and the electro-mechanical models stem from the change in buffering scheme adopted: in the coupled formulation, the combined troponin-calmodulin buffering scheme of the ToR-ORd and ORd models is split up into its two components and the Land dynamic formulation for troponin C is adopted. As a consequence, the coupled models now predict that less calcium is bound to these buffers (Fig. 3-B, D, insets), increasing the free calcium availability inside the cell. Overall, the agreement of simulated behaviour and experimental data reported in this section provides a reliable basis for the credibility of the calibrated coupled models.

### 3.2 Cellular simulations of transmural heterogeneity in human cardiac electro-mechanics

**Fig. 4:**
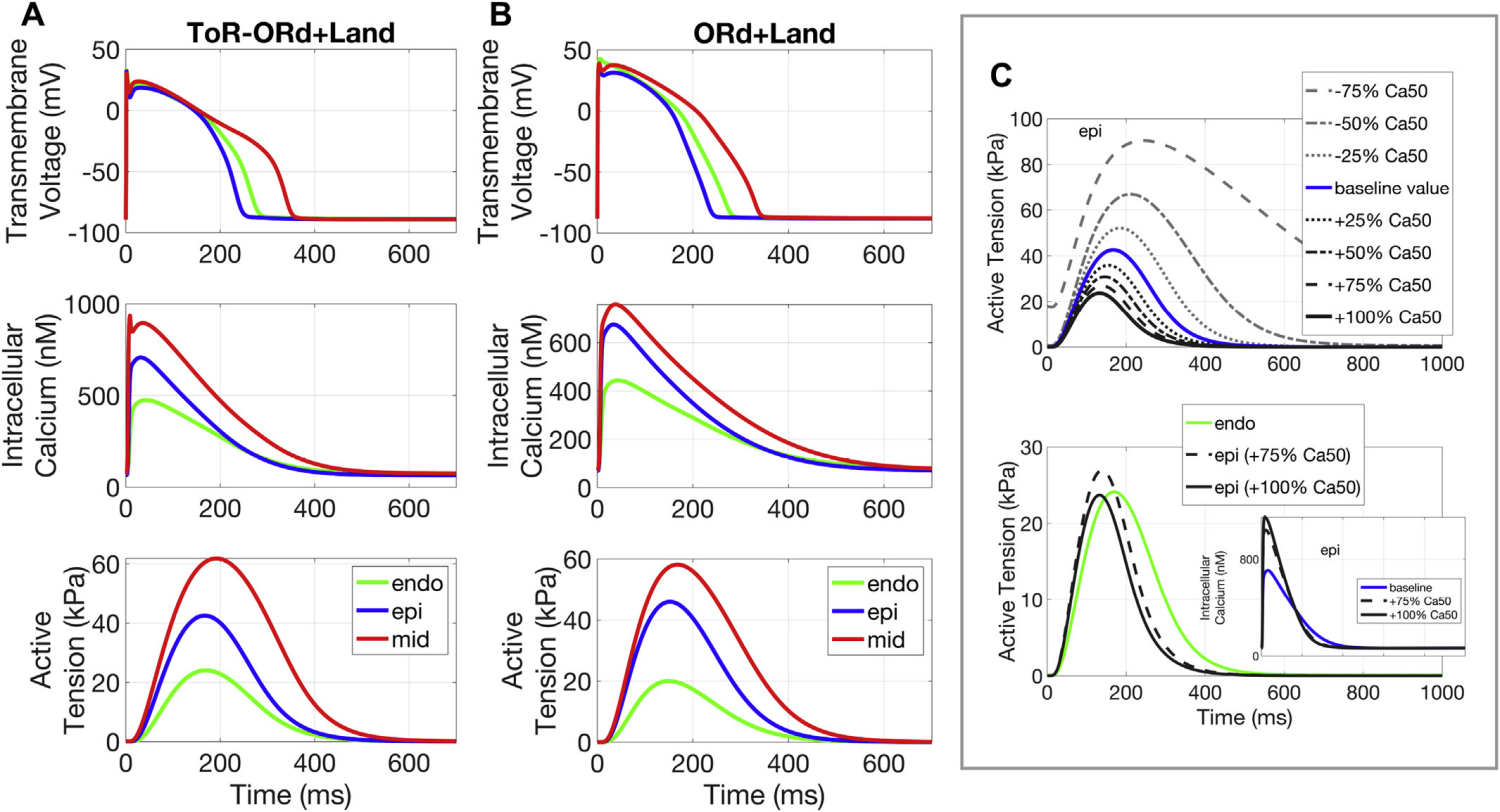
**Transmural heterogeneity effects in electro-mechanical function.** Single cell simulation results under heterogeneous electrophysiological transmural properties. A-B: Comparison between ToR-ORd+Land (A) and ORd+Land (B) models in AP, CaT, and active tension for endocardial, epicardial, and mid-myocardial cells. C: Active tension development in epicardial cells can be additionally modulated by myofilament calcium sensitivity (Ca50 value). Sensitivity analysis results predict that epicardial calcium sensitivity in the range between 1.75x and 2x the baseline Ca50 value (i.e. reduced sensitivity) achieves a similar active tension for epicardial compared to endocardial cells, as experimentally reported by Haynes et al. (2014). Inset: changes in CaT induced by calcium sensitivity.

Simulation results with the calibrated cellular electro-mechanical models, ToR-ORd+Land and ORd+Land, are able to replicate the transmurally varying features of healthy human cardiac electro-mechanics in terms of AP repolarisation, CaT decay sequences (as reported by Lou et al. (2011)), and isometric tension (as reported by Haynes et al. (2014)). These include the well-known transmurally heterogeneous behaviour of AP repolarisation (Fig. 4-A and 4-B, first row), shown to be a compensatory mechanism for the spatially-induced delays in epicardial activation, in order to achieve a more transmurally synchronous contraction (Boukens et al., 2015). Mid-myocardial cells experience the longest APs, followed by endocardial and epicardial cells. The same transmural heterogeneity was found in the CaTs (Fig. 4-A and 4-B, second row), and also, consequently, in the active tension transients (Fig. 4-A and 4-B, third row).

The simulation results show a significantly higher tension in the mid-myocardium compared to the epicardial and endocardial cells, which is consistent with previous experimental findings (Haynes et al., 2014). However, the simulations also predict a higher tension in the epicardium than in the endocardium (Fig. 4-A and 4-B, third row), while experimental data indicate similar values for both cell types. The experimental data by Haynes et al. (2014) also suggest that the endocardium may present a higher calcium sensitivity compared to the epicardium, which has not been considered thus far in our simulations. To test this hypothesis, we performed a sensitivity analysis exploring the range of 0 to 2-fold changes in calcium sensitivity for the epicardial cells.

Results in Fig. 4-C indicate that a decrease in epicardial calcium sensitivity (between 1.75x and 2x the baseline Ca50 value) recovers a peak active tension in epicardial cells similar to that found in the endocardial ones, consistent with the experimental data. This also leads to a faster epicardial onset of contraction, time to peak, and faster relaxation rate compared to endocardium, as reported by Cordeiro et al. (2004) for canine samples. Such changes lead also to higher CaT amplitudes (Fig. 4-C, inset), as calcium sensitivity modulates the amount of calcium bound to troponin C.

### 3.3 Simulation of inotropic and arrhythmogenic effects of multichannel action reference compounds

*Dofetilide*. Dofetilide (almost) selectively blocks IKr. It induces a dose-dependent increase in the QT interval, and TdP is the most dangerous side effect of this therapy. In this study, Dofetilide concentrations were explored in the range of 0.02–0.1 μM, in correspondence with experimental investigations (Guo et al., 2011; Page et al., 2016; Nguyen et al., 2017).

**Fig. 5:**
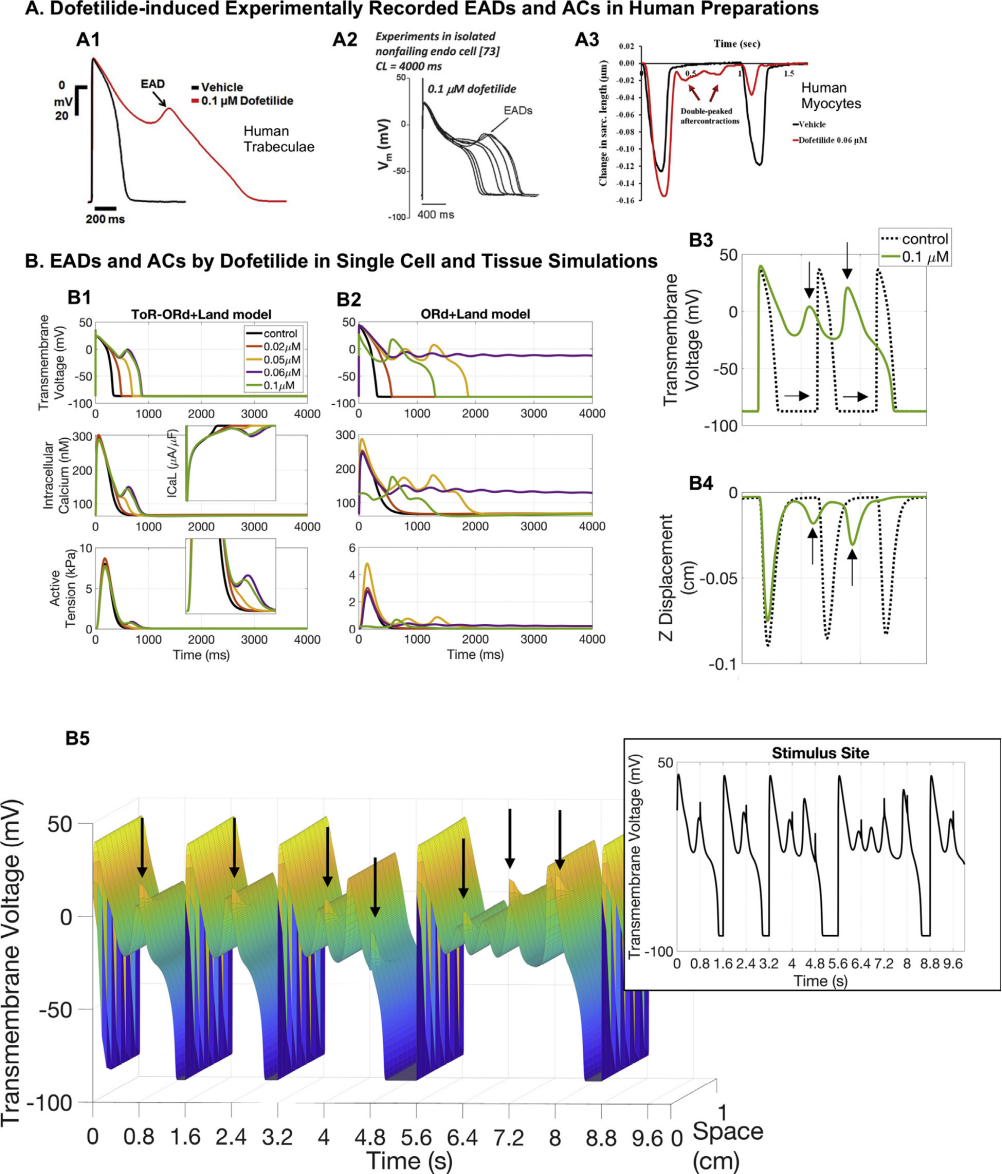
**Dofetilide.** A: Experimental evidence of Dofetilide-induced abnormalities recorded in human trabeculae at 1 Hz pacing (A1: EADs, figure reproduced with permission from Page et al. (2016)) and human myocytes paced at 0.25 Hz (A2: EADs, figure reproduced from O’Hara et al. (2011) as allowed by the CC-BY licence) and 1 Hz (A3: aftercontractions, figure reproduced from Nguyen et al. (2017) as allowed by the CC-BY licence). B: Single cell and tissue simulations of Dofetilide exposure. B1-B2: Single cell comparison between ToR-ORd+Land (B1) and ORd+Land (B2) model predictions. Dofetilide-induced AP prolongation favours a persistent ICaL current, which mediates positive inotropic effects at low doses and EADs formation for the highest ones. B3-B5: 3D tissue simulations at 1.25 Hz pacing. 0.1 μM of Dofetilide induces EADs (B3) that trigger aftercontractions (B4). Contractility escapes indicated by horizontal arrows. AP and z displacements recorded at the free surface opposite stimulation. B5: AP time course in a line connecting two opposite corners. Arrows highlight APs propagation failure. The inset reports the AP time course at the stimulus site. Results in B3-B5 obtained with the ORd+Land model.

Simulations conducted with the cellular ToR-ORd+Land model at the lowest drug concentration (0.02 μM) show that Dofetilide prolongs the AP, which in turn induces an increase in intracellular calcium, and therefore of active tension (pacing frequency of 0.25 Hz, Fig. 5-B1). For higher concentrations, Dofetilide is capable of inducing repolarisation abnormalities in the form of EADs due to ICaL reactivation, resulting in the triggering of aftercontractions at the highest concentrations tested (0.06 and 0.1 μM, pacing frequency of 0.25 Hz, Fig. 5-B1). Dofetilide-induced EADs at 0.1 μM (0.25 Hz pacing) have been experimentally reported in human cardiomyocytes (Fig. 5-A2, Guo et al., 2011), and also at 1 Hz pacing in human trabeculae (Fig. 5-A1, Page et al., 2016). Data from human cardiomyocytes have also shown aftercontractions at 0.06 μM at 1 Hz pacing frequency (Fig. 5-A3, Nguyen et al., 2017). Consistent with the ToR-ORd+Land model, simulations with the ORd+Land model also show that APD prolongation induces a positive inotropic effect at the lowest concentration tested (pacing frequency of 0.25 Hz).

Simulations with the ORd+Land model, however, predict an earlier onset of Dofetilide-induced aftercontractions already at a low concentration of 0.05 μM. Complete repolarisation failure also occurs at higher Dofetilide doses in the ORd-Land model, highlighting the higher sensitivity of the ORd model to IKr current blockage compared to the more recent ToR-ORd model.

Dofetilide-induced EADs, as observed in isolated cells, were also replicated in tissue simulations, as shown in Fig. 5-B3 (0.01 μM Dofetilide, 1.25 Hz pacing). EADs formation in human trabeculae exposed to 0.1 μM Dofetilide at 1 Hz pacing have been previously reported (Fig. 5-A1, Page et al., 2016). In tissue simulations, a significant portion of paced APs at the stimulus site (Fig. 5-B3, dashed traces) failed to propagate due to the formation of EADs and delayed repolarisations, causing contractility escapes (Fig. 5-B3, horizontal arrows). AP propagation failure is also illustrated in Fig. 5-B5 where we report the time course of the transmembrane voltage in a tissue line connecting two opposite corners of the tissue. Contractility escapes have been previously observed in 17% of recordings in human preparations exposed to high Dofetilide concentrations (Nguyen et al., 2017).

The ToR-ORd+Land also demonstrated contractility escapes within the tested range of Dofetilide concentrations (Fig. S5).

**Fig. 6:**
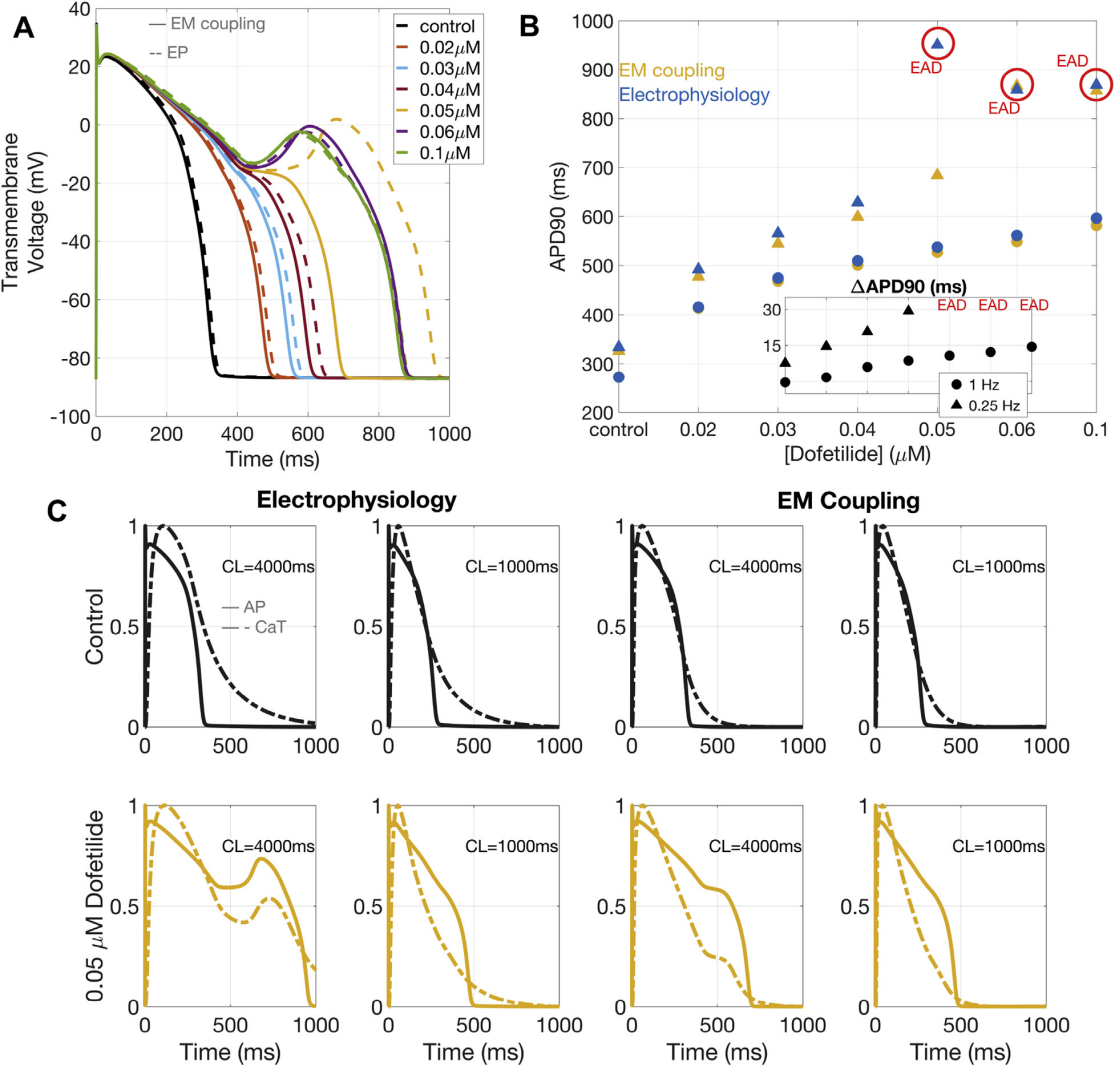
**Electro-mechanical coupling and repolarisation.** Electro-mechanical coupling (EM) mitigates the APD prolongation induced by Dofetilide, and delays EADs onset (A-B). This is mediated by a faster CaT decay under EM coupling (C), which recovers almost entirely during AP repolarisation. Results obtained with the ToR-ORd+Land model. CL: cycle length.

Our simulation results allow us to identify important consequences of electro-mechanical coupling in the myocardial response to Dofetilide exposure. Importantly, electro-mechanical coupling provides a more robust repolarisation compared to electrophysiology-only models (Fig. 6-A and 6-B). Electro-mechanical coupling mitigates the APD prolongation induced by Dofetilide, and also raise the concentration required for the onset of EADs to 0.06 μM. These phenomena are stronger at lower pacing frequencies, but also present at 1 Hz pacing (Fig. 6-B).

Dofetilide-induced APD90 prolongation in the electro-mechanical (ToR-ORd+Land and ORd+Land) and their respective electrophysiology-only (ToR-ORd and ORd) models (for both 0.25 and 1 Hz pacings) is reported in Fig. 6-B. Computed differences in APD90 between the electro-mechanical (ToR-ORd+Land and ORd+Land) and their respective electrophysiology-only (ToR-ORd and ORd) models (for both 0.25 and 1 Hz pacings) are reported in the inset of Fig. 6-B. The explanation for the more robust repolarisation under electro-mechanical coupling is presented in Fig. 6-C, which shows the AP and CaT with electrophysiology versus electro-mechanical models, at 0.25 and 1 Hz pacing, for control conditions and 0.05 μM of Dofetilide. Under electro-mechanical coupling, the CaT decays almost completely before the end of the AP repolarisation. For both coupled and uncoupled models, Dofetilide-induced AP prolongation increases calcium influx into the cell through a persistent ICaL. However, in the presence of electro-mechanical coupling, this effect occurs when the CaT has decayed almost completely, resulting in a smaller chance of inducing additional AP prolongation or EADs.

*Verapamil*. Verapamil blocks both IKr and ICaL, with minor effects on APD and QT interval. Verapamil has a TdP risk of 0 (not classified).

**Fig. 7:**
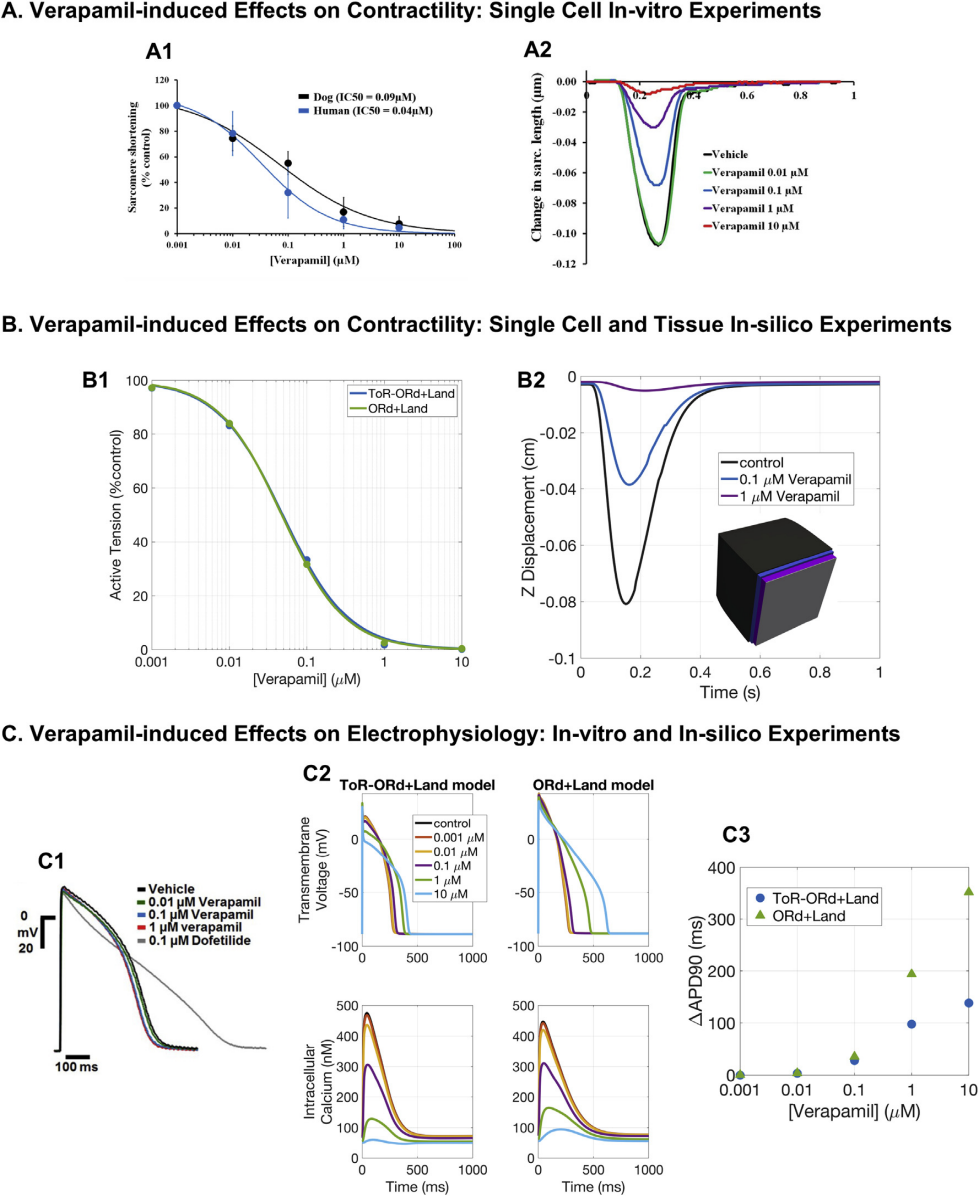
**Verapamil.** A: Experimental evidence of Verapamil-induced effects on cell shortening (figures reproduced from Nguyen et al. (2017) as allowed by the CC-BY licence). B: Single cell and tissue simulation of Verapamil effects. B1: Dose-response curves of peak active tension for Verapamil, using the ToR-ORd+Land and ORd+Land models. B2: Longitudinal displacement of the free face computed in tissue slabs exposed to different drug concentrations. Inset: superimposed tissue slabs at maximal shortenings (undeformed tissue configuration in grey). C: In-vitro (C1) and in-silico (C2-C3) experiments of Verapamil-induced effects on electrophysiology. C1: Human trabeculae (figure reproduced with permission from Page et al., 2016). C2: Verapamil-induced effects on AP and CaT for ToR-ORd+Land and ORd+Land models. C3: APD90 prolongation under Verapamil exposure with respect to control APD90 for both models.

Fig. 7-B1 illustrates simulated dose-response curves of peak active tension at different Verapamil concentrations (0.001, 0.01, 0.1, 1, 10 μM) at 1 Hz pacing, using the ToR-ORd+Land and ORd+Land models. Our results correctly reproduce Verapamil-induced effects on contractility, in agreement with reports classifying it as a negative inotropic compound (Ritchie et al., 2006; Nguyen et al., 2017). Both models predicted a very similar negative inotropic response of human cardiomyocytes to Verapamil. Estimated IC50 and Hill coefficients for active tension reduction resulted in an equal IC50 of 0.05 μM for both models, and Hill coefficients of 1.1 and 1 for ORd+Land and ToR-ORd+Land, respectively. We qualitatively compared these results to experimental dose-response curves of sarcomere shortening by Verapamil in human cardiomyocytes (Fig. 7-A1), which reported an IC50 of 0.04 μM. Moreover, simulations on tissue slabs allowed for the quantification of Verapamil-induced changes in longitudinal displacement (Fig. 7-B2) and showed further agreement with experimental data (Fig. 7-A2).

Despite the very similar inotropic response, mediated by a similar effect on CaT, the ToR-ORd+Land and the ORd+Land models provide different predictions in terms of APD prolongation (Fig. 7-C2). The ORd+Land model produces a much larger APD prolongation at the two highest drug concentrations (of 194 and 353 ms) than the ToR-ORd model (of 98 and 138 ms), see Fig. 7-C3. Disagreements between experimental (Page et al., 2016) and in-silico predictions of Verapamil-induced APD prolongation have been reported before (Britton et al., 2017a) and ascribed to either an incorrect balance between the IKr and ICaL currents in the ORd model, or an overestimation of the IC50 for IKr block measured in voltage clamp experiments. Thanks to its revised IKr and ICaL formulations, the ToR-ORd+Land model predictions are closer to experimental data, which report no APD prolongation (Fig. 7-C1), than those of the ORd+Land model. However, the strong dependence of the in-silico predictions on the IC50 data used as input inevitably persists.

Additional analyses with the ToR-ORd+Land model show that, in order to reproduce experimental data on APD, IC50 for IKr has to be one order of magnitude bigger (considering the IC50 for ICaL of 0.2 μM and Hill coefficient of 0.8, as done in this study). In that scenario, we observe a mild APD shortening (maximum shortening of 10 ms) for concentrations ranging between 0.001 and 1 μM, while 10 μM still yields APD prolongation (76 ms). It is worth noting that such a high concentration of 10 μM (more than 100-fold the maximal effective free therapeutic concentration) has not been tested in experiments. However, prolonged QT and QTc intervals (from 375 ± 24 ms to 469 ± 54, and from 440 ± 30 to 511 ± 44 ms, respectively) have been reported in cancer patients under high doses of Verapamil infusion (De Cicco et al., 1999).

*Quinidine*. Quinidine is a class I antiarrhythmic agent that acts as a blocker of voltage-gated sodium channels and is used to prevent ventricular arrhythmias in spite of safety concerns, as it also blocks voltage-gated potassium channels. Quinidine is known to have a negative inotropic effect (Hoffmeister et al., 1987). Importantly, according to published IC50 data (Kramer et al., 2013), Quinidine also blocks significantly ICaL, leading to a weaker contraction of cardiomyocytes.

**Fig. 8:**
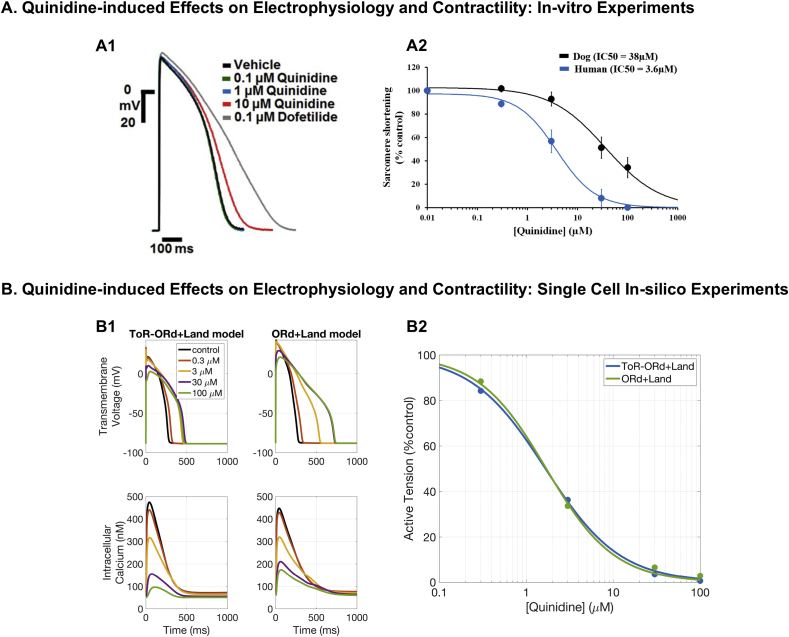
**Quinidine.** A: Experimental evidence on Quinidine-induced effects on human AP (A1, figure reproduced with permission from Page et al., 2016) and cell shortening (A2, figure reproduced from Nguyen et al. (2017) as allowed by the CC-BY licence). B: Single cell simulations of Quinidine effects. B1: Comparison between the ToR-ORd+Land and ORd+Land models in response to Quinidine action in terms of AP and CaT. B2: Quinidine dose-response curves for active tension reduction for both the ToR-ORd+Land and ORd+Land models.

As shown both in-silico (Fig. 8-B2) and by experimental data from human cardiomyocytes (Fig. 8-A2), Quinidine generates a dose-dependent decrease in both active tension and sarcomere shortening, indicating a strong inotropic effect of the drug, which is already present at therapeutic doses. At 1 Hz pacing, IC50 for active tension was 1.7 μM with a Hill coefficient of 1.1 for the ORd+Land, and IC50 of 1.7 μM and Hill coefficient of 1 for ToR-ORd+Land, compared to an experimental IC50 for sarcomere shortening of 3.6 μM. The small differences in the in-silico predictions of Quinidine-induced inotropic effects can be ascribed to the differences between the ToR-ORd and ORd models. In particular, one of the most relevant features of the recent ToR-ORd model compared to ORd is its capability of correctly reproducing the negative inotropic effect of sodium block, mediated by reduced intracellular calcium (Tomek et al., 2019). In the context of Quinidine exposure, the negative inotropic effect associated with ICaL inhibition is therefore further increased by sodium block, which explains the different predictions between the two coupled models (Fig. 8-B2).

As per Verapamil, disagreement in APD prolongation between model predictions and experimental results across different concentrations, IC50 datasets, and hERG block models has been previously noted (Britton et al., 2017a). Simulations of Quinidine with the ORd model predicted a larger APD prolongation and triangulation increase than experimentally observed, as well as the predicted occurrence of repolarisation abnormalities not present in experiments. Simulation results with the ToR-ORd+Land model showed an APD prolongation of 39, 169, 202, and 185 ms at Quinidine doses of 0.3, 3, 30, and 100 μM, respectively (Fig. 8-B1). Significant prolongation of APD has been also experimentally reported at high Quinidine doses, although starting around 10 μM (Fig. 8-A1). These differences can be ascribed to the reported IC50 of 0.72 μM (Hill coefficient of 1.06) for IKr, which implies very high IKr block at the doses considered, starting from 28% at 0.3 μM and already 82% at 3 μM.

## 4 Discussion

In this study, we present strongly-coupled electro-mechanical models of human ventricular myocytes and myocardial tissue, by integrating existing human models of cellular electrophysiology and contractility. Particularly, we thoroughly compare the behaviour of the new ToR-ORd model with its predecessor ORd model, both coupled with the Land model. The models are calibrated to yield active contraction properties that are physiological. Independent validation of the electro-mechanical behaviour of these models show that they are able to reproduce the transmural heterogeneities of excitation-contraction coupling and force generation seen in human experimental data, and also correctly predict the inotropic response of different multichannel action reference compounds. The simulations are able to explain the partial mismatch between in-silico and in-vitro predictions of drug-induced APD prolongation for multichannel action compounds. They also show that models with electro-mechanical coupling have better cellular repolarisation robustness under drug exposure than electrophysiology-only models. Electro-mechanical coupling mitigates the APD prolongation induced by Dofetilide, and also delays the onset of EADs formation. Overall, this study provides a human-based mechanistic framework for future investigations into the complex interplay between the electrical and mechanical substrates of cardiac function and its modulation under pharmacological action. It paves the way for extensions and translations of the results towards tissue- and organ-level computational studies of cardiac patho-physiology.

### 4.1 Human electro-mechanical cell models

The electro-mechanical simulations conducted in this study feature the use of two different human electrophysiology models, the ToR-ORd model, and its predecessor ORd. Both models are coupled with the Land model and provide descriptions of human cardiomyocyte electro-mechanical behaviours. Such descriptions are in agreement with the AP biomarkers (Britton et al., 2017b) considered in this study (time to peak, APD50, and APD90). However, as explained in Tomek et al. (2019), the ToR-ORd model provides an improved description of ventricular electrophysiology, in particular in terms of AP plateau, dynamics of accommodation of the APD to heart rate acceleration, and sodium-block induced changes in intracellular calcium.

Calcium (Coppini et al., 2013) and active tension (Mulieri et al., 1992; Pieske et al., 1996; Rossman, 2004) human data are also reproduced by the models.

We have shown a strong agreement between the simulated CaT biomarkers with the new coupled models and the experimental data reported in Coppini et al. (2013). However, such data differ from the ones presented in Piacentino et al. (2003) and Høydal et al. (2018), especially for the time to peak. There is substantial experimental variability in the CaT characterization shown in the aforementioned studies. Possible reasons behind that could be related to the type of preparations, the isolation procedure (Yue et al., 1996; Voigt et al., 2015), and the recording techniques.

The electro-mechanical models in this study are constructed from models featuring components either human-specific or reparametrised from animals. They are therefore the result of significant and ongoing research (see for instance, Trayanova and Rice, 2011, Balakina-Vikulova et al. (2020)).

The human-relevant focus will facilitate future research on disease and drug-induced effects, where the translation from preclinical to clinical findings is particularly challenging due to species-specific differences (Perel et al., 2007).

Both coupled models feature a dynamic description of calcium binding to troponin C that links the electrophysiology and contractility subcomponents and allows a more accurate prediction of free intracellular calcium availability (Coppini et al., 2013) compared to previous steady-state buffers used in the ToR-ORd and ORd models. The importance of modelling the dynamic intracellular calcium buffering in order to reproduce experimental data has been noted before (Ji et al., 2015).

However, when comparing the two models, we observe that the ToR-ORd model provides a more robust prediction of the drug-induced effects as demonstrated by our simulation results for Dofetilide and Verapamil, in addition to the previously reported model’s improvements with respect to the ORd model (Tomek et al., 2019). The ToR-ORd model should therefore be preferred to the ORd model for future human-relevant drug and disease studies.

### 4.2 Transmural heterogeneity in cellular electro-mechanical properties

Numerous studies have addressed ionic heterogeneity within the human ventricular myocardium (Li et al., 1998; Gaborit et al., 2007), and heterogeneities of excitation-contraction coupling and force generation in animal hearts (Cordeiro et al., 2004; Stelzer et al., 2008; van der Velden et al., 2011). However, the role of spatial heterogeneities of excitation-contraction coupling, intracellular calcium handling, and force generation in the human heart remains unclear (Lou et al., 2011). Potentially, the presence of those heterogeneities may imply that both physiological contractile properties and susceptibility to pathological remodelling during disease differ across the ventricular wall.

The electro-mechanical models presented in this study incorporate heterogeneous ionic currents, fluxes, and buffers distributions based on the datasets published in Tomek et al. (2019) and O’Hara et al. (2011). We show that heterogeneous subcellular electrophysiology properties are reflected by similar heterogeneities at the level of cellular function. Cellular AP and CaT transmural differences resemble the data presented in Lou et al. (2011). Such heterogeneities are known to be larger in isolated preparations compared to the electrotonically-coupled ventricular myocardium (Taggart et al., 2001). Heterogeneous CaTs translate into heterogeneous active tensions, following the well-known physiological relationship between intracellular calcium and force development (Sun and Irving, 2010). Prediction of active tension development in the mid-myocardial cell is in agreement with experimental data, which show that they produce the highest tension. However, to fully recapitulate the experimental dataset on heterogeneous active tensions (Haynes et al., 2014), our sensitivity analysis shows that heterogeneities in myofilament calcium sensitivities also need to be taken into account. This is in accordance with the experimental report on non-failing human samples exhibiting a statistically significant increase in calcium sensitivity going from sub-epicardial to sub-endocardial regions (Haynes et al., 2014).

Khokhlova et al. (2018b) had already suggested the need to consider endo/epi differences in cooperativity of calcium activation of myofilaments to replicate experimental results on the distinct cellular responses to changes in mechanical load. They also suggested that mechano-calcium-electric feedback is key to modulate the electrophysiological and contractile properties of myocytes across the ventricular wall (Khokhlova et al., 2020).

Experimental data on transmural heterogeneity on CaT and mechanical activity are controversial. In particular, Wan et al. (2005) showed a lower amplitude of the CaT in the endo cells of guinea pig left ventricles than in the epi cells, consistent with our findings, whereas others reported that in cardiomyocytes of rabbit, dog, and human no significant differences were found (as reviewed by Khokhlova et al., 2018a).

Several studies have linked cellular and sub-cellular heterogeneities to more macroscopic functional end-points and provided context to the heterogeneous mechanical functions observed in this study. There are clinical studies that have stated that transmural variation in the contractile function of the human myocardium may influence clinical end-points (de Simone et al., 1996; Wachtell et al., 2010). A few computational studies demonstrated the importance of accounting for heterogeneous distributions of contractile properties in order to replicate experimental evidence (Wang et al., 2016; Khokhlova et al., 2017; Vaverka et al., 2018). A very recent study (Dusturia et al., 2019) has also shown that a thicker mid-myocardium layer may improve pumping efficacy and contractility.

Our study was designed to investigate the role of well-documented transmural electrophysiological heterogeneities in the human myocardium and their impact on the mechanical contraction. We investigated whether such sub-cellular electrophysiological changes could explain the cellular level experimental data reported for human samples from different ventricular regions. Indeed, we showed that an additional source of transmural heterogeneity was needed to fully recapitulate the experimental evidence. However, we did not investigate the effect of other sources of heterogeneity reported in the literature such as transmural distribution of fast and slow isomyosins (Eisenberg et al., 1985; Litten et al., 1985; Reiser et al., 2001; Carnes et al., 2004).

### 4.3 In-silico predictions of inotropic and pro-arrhythmia liabilities

Pro-arrhythmic and inotropic liabilities are major concerns for the pharmaceutical industry (Laverty et al., 2011), and it is highly recommended to test the potential of new candidate drugs to induce changes in cardiac electrophysiology and contractility early in the drug discovery pipeline (Nguyen et al., 2017). In-silico approaches represent a human-relevant fast and cheap way of conducting such screenings and have already shown their potential to accurately predict clinical risk (Passini et al., 2017, 2019; Li et al., 2019). However, computational approaches for inotropic risk prediction are still lacking. Here, we present a modelling and simulation framework to investigate simultaneously drug-induced effects on myocardial contractility and pro-arrhythmia. The simulations of drug-induced effects on human electro-mechanical function conducted in this study correctly classify negative and positive inotropic reference compounds and explain the mechanisms behind the observed effects on contractility. We also show improved agreement between in-vitro and in-silico predictions on APD prolongation compared to previous studies (Britton et al., 2017a).

The Dofetilide-induced inotropic response predicted by our models closely resembles the experimental data from Nguyen et al. (2017), including the occurrence of aftercontractions at high concentrations. The mechanism underpinning such response is related to the APD prolongation due to IKr block, and subsequent intracellular calcium increase. This is in good agreement with previous reports on the interaction between calcium handling and APD (Tande and Refsum, 1990). The aftercontractions triggered in single cell by EADs can also be replicated in tissue simulations. This is in further agreement with experimental recordings under Dofetilide action in both human cardiomyocytes (Nguyen et al., 2017) and trabeculae (Page et al., 2016). Contractility escapes, i.e. when electrical stimuli do not result in a contraction transient, are also present in both in-silico and in-vitro experiments (Nguyen et al., 2017).

Additional evidence on the key role of intracellular calcium in determining the magnitude of cardiac contraction is provided by our simulation results for Verapamil and Quinidine. For both compounds, we predict a concentration-dependant decrease in active tension, confirmed by experimentally recorded decrease in sarcomere shortening in human cardiomyocytes (Nguyen et al., 2017). As a consequence, Verapamil is in fact generally avoided in patients with severe left ventricular dysfunction and our simulation results confirmed this potential risk by demonstrating the drug’s negative inotropic effects.

Both Verapamil and Quinidine are multichannel action compounds that block, among other currents, both IKr and ICaL. The IKr block, which is significant even for low concentrations of these two drugs, determines the AP prolongation, while the ICaL block affects primarily the CaT. Our simulations show that an IKr-only block leads to a mild increase in calcium followed by a positive inotropic effect. This is clearly counteracted by the direct calcium block under Verapamil or Quinidine exposure. On the other hand, an ICaL block alone leads to a mild AP shortening which, for Verapamil and Quinidine, is opposed by the IKr block.

The prediction of APD changes for multi-channel action compounds is a very challenging task (Britton et al., 2017a), due to concomitant effects on both inward and outward currents. We show that the new ToR-ORd+Land model is more robust against APD prolongation under Verapamil exposure, while still predicting a longer prolongation than experimental data (Page et al., 2016). This is an inevitable consequence of the reported IC50 values available, inducing very high levels of IKr block. Our simulations predict that, in order to replicate the experimental data, the IC50 for IKr has to be one order of magnitude bigger. This is particularly important in light of the recent heightened awareness of high variability in IC50 measurements (Li et al., 2020). Experimental data report both APD prolongation (Amerini et al., 1985; Kimura et al., 1992) and shortening (Page et al., 2016; Lei et al., 2017) induced by Verapamil and additional studies are needed to overcome such controversy.

In-vitro experiments under Quinidine exposure report no APD prolongation except for the highest dose of 10 μM (Page et al., 2016). Our simulation results predict APD prolongation starting from 3 μM with a significant reduction in peak potential.

### 4.4 Electro-mechanical coupling and repolarisation

In our in-silico analyses of the effects of Dofetilide on electro-mechanical function (in both cardiomyocytes and trabeculae) we observe a more robust electrical repolarisation in the presence of the electro-mechanical coupling, compared to electrophysiology-only models. While consistent for different pacing frequencies, this is more evident at slow pacing. We are able to explain such behaviour by detailed analyses of the time course of the simulated AP and CaT. Coupling-induced changes on the CaT allow for calcium to decay almost completely within the duration of the AP. Under such circumstances, the APD prolongation induced by drug exposure has less opportunity to translate into calcium abnormalities. This can be also seen in terms of the electro-mechanical window (EMw), defined as the difference between the duration of electrical and mechanical systole and computed in previous studies as the difference between the APD and CaT duration at 90% repolarisation/decay (Passini et al., 2019). In baseline conditions, the EMw is shorter in electro-mechanical models than in electrophysiology-only models. However, the EMw shortening induced by Dofetilide, known to be a pro-arrhythmic effect of IKr blockers, is greater in electrophysiology-only models than in electro-mechanical ones (Supplementary Figs. S4–A). As shown in Passini et al. (2019), the shortening of the EMw is an effective biomarker of TdP prediction, and it can be used here to explain the higher propensity of electrophysiology-only models to develop repolarisation abnormalities.

### 4.5 Limitations and future work

While the models presented in this study have been able to recapitulate some of the transmurally varying electro-mechanics features of the healthy human myocardium, additional sources of heterogeneity have been reported in the literature. Inclusion of these aspects of heterogeneity in the models would provide a more comprehensive description of the heterogeneous cardiac substrates. As suggested in previous studies (Eisenberg et al., 1985; Litten et al., 1985; Reiser et al., 2001; Carnes et al., 2004) myosin expression may differ across the myocardium, and further simulations are needed to assess the impact of a transmural gradient in such properties on cardiac function.

Another key limitation of this study is the lack of after-load simulations. Contractility response is critically affected by pre-load and after-load conditions and their simulations, together with isometric ones, would provide a more exhaustive description of contraction modes. Further model development is needed to include a mathematical representation of the dynamic changes in sarcomere length. Evaluation of such simulation results would also require human-relevant data currently unavailable.

Finally, a key factor to consider for the comparisons reported in this study between experimental and simulation data is the differences between setups. In particular, experiments are often conducted at a fixed muscle length, which would allow some sarcomere shortening even in isometric contractions (de Tombe and ter Keurs, 2016), whereas in our simulations we can only control sarcomere length by means of extension ratios. The 3D simulation framework presented in this study can be key in overcoming this limitation, combining cellular active tension with tissue deformation and therefore providing a closer representation of the experimental setup.

## 5 Conclusion

In this study, we integrated the most recent models of human ventricular electrophysiology and active contraction and calibrated them using human experimental data. This process yielded physiological simulated active tension, AP, and CaT in agreement with experiments obtained in human preparations. Furthermore, the simulated electro-mechanical function reproduced the reported transmurally heterogeneous properties of human myocardium.

We quantified the effect of the electro-mechanical coupling on electrophysiology and force generation in virtual human ventricular cardiomyocytes and myocardial tissue, both in control conditions and under drug exposure. Specifically, we correctly predicted the inotropic response of different multichannel action reference compounds. Furthermore, we generated additional evidence to explain the partial mismatch between in-silico and in-vitro predictions of drug-induced APD prolongation. Simulations demonstrated that cardiac electro-mechanical coupling provides a more robust repolarisation under drug exposure compared to electrophysiology-only models.

The human-based mechanistic framework presented in this study will enable future investigations into the complex interactions between electrophysiology and contractility, also allowing the simultaneous assessment of pro-arrhythmia and inotropic liabilities of pharmacological therapies.

## Author statement

Francesca Margara: Conceptualization, Methodology, Formal analysis, Software, Writing - Original Draft, Writing - Review & Editing. Zhinuo J. Wang: Conceptualization, Methodology, Software, Writing - Original Draft, Writing - Review & Editing. Francesc Levrero-Florencio: Methodology, Software, Writing - Review & Editing. Alfonso Santiago: Software, Writing - Review & Editing. Mariano Vázquez: Software, Writing - Review & Editing. Alfonso Bueno-Orovio: Conceptualization, Methodology, Writing - Original Draft, Writing - Review & Editing, Supervision. Blanca Rodriguez: Conceptualization, Methodology, Writing - Original Draft, Writing - Review & Editing, Funding Acquisition, Resources, Supervision.

## Funding sources

This work was funded by the Personalised In-Silico Cardiology (PIC) project (10.13039/100010661European Union’s Horizon 2020 research and innovation programme under the Marie Sklodowska-Curie grant agreement 764738); a 10.13039/100010269Wellcome Trust Fellowship in Basic Biomedical Sciences to B.R. (214290/Z/18/Z), a 10.13039/501100000274British Heart Foundation (BHF) Intermediate Basic Science Fellowship to A.B. (FS/17/22/32644), the CompBioMed 1 and 2 Centre of Excellence in Computational Biomedicine (10.13039/100010661European Commission Horizon, 2020 research and innovation programme, grant agreement No. 675451, and No. 823712), an NC3Rs Infrastructure for Impact Award (NC/P001076/1), the TransQST project (Innovative Medicines Initiative 2 Joint Undertaking under grant agreement No 116030, receiving support from the 10.13039/100010661European Union’s Horizon 2020 research and innovation programme and 10.13039/100013322EFPIA), and the Oxford BHF Centre of Research Excellence (RE/13/1/30181). The authors gratefully acknowledge computational resources provided by a PRACE project (2017174226) and an Amazon Web Services (Machine learning research award, 364348137979).

## Data statement

Code and supporting research data are available under request.

## Declaration of competing interest

None.
