# Supplementary material for: In-silico human electro-mechanical ventricular modelling and simulation for drug-induced pro-arrhythmia and inotropic risk assessment
Source: Prog Biophys Mol Biol. 2021 Jan;159:58–74. doi: 10.1016/j.pbiomolbio.2020.06.007 (PMC7848595; doi:10.1016/j.pbiomolbio.2020.06.007)
Supplement: Multimedia component 1 [file mmc1.docx]

# Supplementary Information

# Supplementary Material

## **S1: Electro-mechanical coupling**

The total calcium concentration present in the cell (${[{Ca}^{2+}]}_{total}$) can be decomposed as the sum of intracellular free calcium (${[{Ca}^{2+}]}_{i}$), calcium bound to calmodulin (CMDN, ${[{Ca}^{2+}]}_{CMDN}$), and to troponin C (TRPN, ${[{Ca}^{2+}]}_{TRPN}$), such that

$${[{Ca}^{2+}]}_{total}= {[{Ca}^{2+}]}_{i}+ {[{Ca}^{2+}]}_{CMDN}+ {[{Ca}^{2+}]}_{TRPN}. (1)$$

A time differentiation of the previous equation yields

$$\frac{d\left[ {Ca}^{2+} \right]_{total}}{dt}= \frac{d\left[ {Ca}^{2+} \right]_{i}}{dt}+ \frac{d\left[ {Ca}^{2+} \right]_{CMDN}}{dt}+ \frac{d\left[ {Ca}^{2+} \right]_{TRPN}}{dt}. (2)$$

Equivalently, changes in total calcium are prescribed by the conservation of calcium ions through the membrane and the sarcoplasmic reticulum, given by:

$$\frac{d\left[ {Ca}^{2+} \right]_{total}}{dt}=\left( -I_{pCa}- I_{Cab}+2I_{NaCa,i} \right)\frac{A_{cap}}{2Fv_{myo}}-J_{up} \frac{v_{nsr}}{v_{myo}}+J_{diff,Ca} \frac{v_{ss}}{v_{myo}}. (3)$$

In the original ToR-ORd and ORd models, the binding of calcium to calmodulin and troponin C buffers is modelled by the algebraic expressions

$${[{Ca}^{2+}]}_{CMDN}= \left[ \bar{CMDN} \right]\frac{\left[ {Ca}^{2+} \right]_{i}}{\left[ {Ca}^{2+} \right]_{i}+K_{CMDN}}, (4)$$

$${[{Ca}^{2+}]}_{TRPN}= \left[ \bar{TRPN} \right]\frac{\left[ {Ca}^{2+} \right]_{i}}{\left[ {Ca}^{2+} \right]_{i}+K_{TRPN}}, (5)$$

and therefore

$$\frac{d\left[ {Ca}^{2+} \right]_{total}}{dt}= \frac{d\left[ {Ca}^{2+} \right]_{i}}{dt}+ \frac{d\left[ {Ca}^{2+} \right]_{CMDN}}{dt}+ \frac{d\left[ {Ca}^{2+} \right]_{TRPN}}{dt} = \frac{d\left[ {Ca}^{2+} \right]_{i}}{dt} \left( 1+ \frac{[\bar{CMDN}]K_{CMDN}}{\left( {[{Ca}^{2+}]}_{i}+K_{CMDN} \right)^{2}}+ \frac{[\bar{TRPN}]K_{TRPN}}{\left( {[{Ca}^{2+}]}_{i}+K_{TRPN} \right)^{2}} \right), (6)$$

yielding

$$\frac{d\left[ {Ca}^{2+} \right]_{i}}{dt}= \beta_{Cai}\left( \left( -I_{pCa}-I_{Cab}+2I_{NaCa,i} \right)\frac{A_{cap}}{2Fv_{myo}}-J_{up} \frac{v_{nsr}}{v_{myo}}+J_{diff,Ca} \frac{v_{ss}}{v_{myo}} \right), (7)$$

where

$$\beta_{Cai}=\frac{1}{1+ \frac{[\bar{CMDN}]K_{CMDN}}{\left( {[{Ca}^{2+}]}_{i}+K_{CMDN} \right)^{2}}+ \frac{[\bar{TRPN}]K_{TRPN}}{\left( {[{Ca}^{2+}]}_{i}+K_{TRPN} \right)^{2}}}. (8)$$

In the ToR-ORd and ORd models, the amount of calcium bound to buffers is therefore described through a combined scheme that includes both troponin C and calmodulin, and provides a steady-state approximation of the phenomenon.

In order to implement the electro-mechanical coupling, the combined buffering scheme was separated, keeping an algebraic formulation for the calmodulin buffer and including a dynamic calcium buffering for troponin C. We defined the amount of calcium bound to troponin C ($\left[ {Ca}^{2+} \right]_{TRPN}$) as the fraction of troponin C units with calcium bound to its regulatory binding site ($CaTRPN$) multiplied by a constant maximum concentration of calcium ions that can bind to troponin C ($\left[ {Ca}^{2+} \right]_{TRPN,max}$ = 0.07 mM, as considered in O’Hara et al., 2011), leading to

$$\frac{d\left[ {Ca}^{2+} \right]_{TRPN}}{dt}=\left[ {Ca}^{2+} \right]_{TRPN,max}\frac{dCaTRPN}{dt}. (9)$$

We then adopted the formulation provided by Land and colleagues (Land et al., 2017) to describe the dynamic binding of calcium to troponin C:

$$\frac{dCaTRPN}{dt}= k_{TRPN}\left( \left( \frac{\left[ {Ca}^{2+} \right]_{i}}{\left[ {Ca}^{2+} \right]_{T50}} \right)^{n_{TRPN}}\left( 1-CaTRPN \right)-CaTRPN \right), (10)$$

where $k_{TRPN}$ represents the unbinding rate, and $n_{TRPN}$ the cooperativity of the calcium- troponin C binding rate. The parameter $\left[ {Ca}^{2+} \right]_{T50}$ describes the half-activation point.

The final formulation for the transient of the free intracellular calcium then becomes

$$\frac{d\left[ {Ca}^{2+} \right]_{i}}{dt}= {\beta^{*}}_{Cai}\left( \left( -I_{pCa}-I_{Cab}+2I_{NaCa,i} \right)\frac{A_{cap}}{2Fv_{myo}}-J_{up} \frac{v_{nsr}}{v_{myo}}+J_{diff,Ca} \frac{v_{ss}}{v_{myo}}- \frac{d\left[ {Ca}^{2+} \right]_{TRPN}}{dt} \right), (11)$$

where

$${\beta^{*}}_{Cai}=\frac{1}{1+ \frac{[\bar{CMDN}]K_{CMDN}}{\left( {[{Ca}^{2+}]}_{i}+K_{CMDN} \right)^{2}}}. (12)$$

## **S2: Model calibration based on human active tension data**

In the calibration process, from each simulation with the electro-mechanical models, the active tension $Ta$ was computed, and the following biomarkers extracted: time to peak (tp), time to 50% decay (rt50), time to 95% decay (rt95), maximum and minimum values. Those were fed into the following cost function (Land et al., 2017):

$$d_{t}=d\left( tp,\left[ 147,172 \right] \right)+d\left( rt50,\left[ 109,125 \right] \right)+d\left( rt95,\left[ 291,377 \right] \right)$$

$$+10d\left( max(Ta),\left[ 15,25 \right] \right)+25min(Ta) (13)$$

where the distances $d$ between each biomarker and the corresponding experimental ranges were computed and summed to obtain the total cost $d_{t}$ which was then minimised. The two weights in the cost function that multiply minimal (25) and maximal (10) active tension were chosen by the authors of the Land model to ensure good activation and relaxation as a first priority and were kept unvaried in our study.

The ranges of biomarkers chosen as target for this calibration procedure are [15-25] for active tension peak (mN/mm^2^), [147-172] for tp (ms), [109-125] for rt50 (ms), and [291-377] for rt95, based on Table 1 and Land et al. (2017). To minimise the cost function, we used the MatLab function *ga* (with default parameters), which finds the minimum of a function using a genetic algorithm. After creating a random initial population, the algorithm generates a sequence of new populations. At each step, the algorithm uses the individuals in the current generation to create the next population

([*https://uk.mathworks.com/help/gads/how-the-genetic-algorithm-works.html*](https://uk.mathworks.com/help/gads/how-the-genetic-algorithm-works.html)).

The model parameters fitted in this procedure are reported in Table S1 and were chosen based on the following assumptions. Firstly, the active tension Land model parameters are 17 in total (Land et al., 2017). Six of them are related to length changes and would not affect model outputs in our isometric setup. The Tref parameter, which is the tension developed at maximal activation for an extension ratio ($\lambda$) equal to 1, would only scale the model output. Therefore it was not considered as a candidate in the sensitivity analysis. All the remaining parameters were varied one-at-a-time for a +/-10% variation to assess their impact on action potential (AP), calcium transient (CaT), and active tension. Those that significantly affected CaT were discarded (n_TRPN_ and Ca50). Of the remaining, K_TRPN_, K_uw_, K_ws_, r_w_, and r_s_ did not exert a significant effect on active tension to be included in the calibration. Of the three remaining parameters, we did not vary TRPN_50_ as it is indicated by the Land model’s authors to have an important length-dependant effect. Since we were not testing length changes, we decided to leave that property unaffected.

Table S1. Model parameters fitted in the calibration process.

|  | ku | nTm |
| --- | --- | --- |
| Parameter Meaning | blocked (B) to unbound (U) myosin binding sites on actin transition rate | Hill coefficient of the cooperative Hill curve between fraction of troponin C units with calcium bound (CaTRPN) and unblocked states 1-B |
| Parameter Range | 0.01-1 | 1.5-2.5 |
| Original Value | 1 | 5 |
| Calibrated ORd+Land | 0.04 | 2.4 |
| Calibrated ToR ORd+Land | 0.021 | 2.036 |

## **S3: Implementation of Transmural Heterogeneity**

We incorporated transmural heterogeneity in ionic properties to generate human mid-myocardial and epicardial cell models from the endocardial version described in this work. As summarised in Table S2, changes to some of the ionic conductances of mid-myocardial and epicardial cells were implemented following the dataset of transmural differences published in O’Hara et al. (2011), and in Tomek et al. (2019). Mid-myocardial L-type calcium permeability was scaled by 1.8 (Dutta et al., 2016).

Table S2. Scaling factors for model implementation of transmural heterogeneity (O’Hara et al., 2011; Tomek et al., 2019).

|  | epi/endo | | mid/endo | |
| --- | --- | --- | --- | --- |
|  | ORd | ToR-ORd | ORd | ToR-ORd |
| GNaL | 0.6 | 0.6 | 1 | 1 |
| Gto | 4.0 | 2 | 4.0 | 2 |
| PCa, PCaNa, PCaK | 1.2 | 1.2 | 1.8 | 1.8 |
| GKr | 1.3 | 1.3 | 0.8 | 0.8 |
| GKs | 1.4 | 1.4 | 1 | 1 |
| GK1 | 1.2 | 1.2 | 1.3 | 1.3 |
| GNaCa_i_, GNaCa_ss_ | 1.1 | 1.1 | 1.4 | 1.4 |
| GNaK | 0.9 | 0.9 | 0.7 | 0.7 |
| GKb | 0.6 | 0.6 | 1 | 1 |
| Jrel_np,inf,_ Jrel_camk,inf_ | 1 | 1 | 1.7 | 1.7 |
| Jup_np_, Jup_camk_ | 1.3 | 1.3 | 1 | 1 |
| CMDN | 1.3 | 1.3 | 1 | 1 |

## **S4: Drug Trials**

The full list of compounds, with a short description, clinical TdP risk, inotropic effect, IC50 and Hill coefficients, concentrations tested and maximal effective free therapeutic concentrations (EFTPCmax) used in this study to simulate drug action can be found in Table S3.

Table S3. List of compounds considered in our study, with a short description, clinical TdP risk, inotropic effect, IC50 and Hill coefficients for the pore block drug models, concentrations tested and EFTPCmax.

|  | Dofetilide | Verapamil | Quinidine |
| --- | --- | --- | --- |
| Description | Antiarrhythmic Class III | Antiarrhythmic Class IV | Antiarrhythmic Class Ia |
| TdP risk | 1 | 0 | 1 |
| Inotropic effect | positive | negative | negative |
| EFTPCmax (µM) | 0.0021 | 0.088 | 3.237 |
| Concentrations tested (µM) | 0.02 0.03 0.04 0.05 0.06 0.1 | 0.001 0.01 0.1 1 10 | 0.3 3 30 100 |
| IC50(h) INaf | 31.9(0.54) | 32.5(1.33) | 14.6(1.22) |
| IC50(h) Ito | 300(1) |  |  |
| IC50(h) ICaL | 201(1) | 0.2(0.8) | 6.4(0.68) |
| IC50(h) IKr | 0.013(1.56) | 0.25(0.89) | 0.72(1.06) |
| IC50(h) IKs | 135(1) |  |  |

# Supplementary Results

## **S5. Effect of calibration on the contractility model components**

Following calibration, we analysed how all the state variables of the Land model changed due to the use of the fitted parameters. Next, we reported the changes in the steady-state calcium-force relationship. The obtained curves were fit using the MatLab curve fitting toolbox to the following modified Hill equation:

$$Ta=\frac{Tmax}{(1+\left( \frac{Ca50}{Cai} \right)^{h})}$$

where Tmax is maximal active tension, Ca50 is the calcium sensitivity, and h is the Hill coefficient.

Figures S1-A, D illustrate the state variables of the contractility model before and after calibration. Results are shown for the ToR-ORd+Land model, but also hold for the ORd-Land model. In particular, as shown in Figure S1-A, the effect of calibration on active tension is attained through changes in the time course of the fraction of blocked myosin sites on actin (state variable B), with more sites becoming unlocked during one cycle. A small fraction of these sites (1%) also remains unblocked at steady-state, while previously they all returned to their blocked state, generating a significantly small residual tension (0.075 kPa, 0.3% of peak active tension). Importantly, this suggests that actomyosin interactions contribute to diastolic stress, as proposed by King et al. (2011) in intact mouse cardiac myocytes. These changes in blocked myosin sites on actin subsequently lead to more force-generating (variable S, Figure S1-B) and pre-power stroke (variable W, Figure S1-C) states available. Figure S1-D also shows that the calcium bound to troponin (feedback mechanism of the coupled electro-mechanical model) is not altered, therefore explaining why the calibration does not change the electrophysiological behaviour of the models.

Furthermore, as a consequence of the modifications in the contractility model, the steady-state relationship of calcium and force is now different (Figure S1-E). The calibrated ToR-ORd+Land model resulted in a calcium sensitivity of 0.55 $\mu$M compared to the previous 0.60 $\mu$M, maximal active tension of 106.8 compared to the previous 119.1 kPa, and Hill coefficient of 2.9 compared to the previous 6.2.

The impact of the calibrated contractility parameters obtained for the endocardial model on mid-myocardial and epicardial cells is explored in Figure S1-F. These yielded less pronounced transmural differences in force development between the three myocardial layers, in better agreement with the experimental data reported by Haynes et al. (2014). This is a joint effect of the new steady-state calcium-force relationship (Figure S1-E), and transmural differences in CaTs. Both epicardial and mid-myocardial CaTs peak display values falling within the less steep part of the steady-state relationship, compared to endocardial CaTs falling within the steepest region. As a consequence, our calibration process leads to comparable values of tension development between layers to those in Haynes et al. (2014), in contrast to the order of magnitude difference predicted between endocardial and mid-myocardial layers by the non-calibrated electro-mechanical models.

Figure S1. Effect of calibration on the contractility model components (results shown for the ToR-ORd+Land model only). The changes in the two parameters fitted in the calibration process directly affect the time course of the state variable B (blocked myosin binding sites on actin, A). This leads to more force-generating (B) and pre-power stroke (C) states available. The calcium bound to troponin variable is unaltered (D). Calibration changes the steady-state relationship of calcium and force (E). Due to such new steady-state relationship the calibration affects endocardial, epicardial, and mid-myocardial cells differently, based on their CaTs and how steep is the steady-state relationship for those calcium levels. F: Effect of the calibrated endocardial parameters on epicardial and mid-myocardial cells.

## **S6. Force-frequency and length-dependence properties of electro-mechanical models**

We illustrate here the force-frequency dependence of the ToR-ORd+Land model in control conditions, by reporting the simulated AP, CaT, and active tension at three different cycle lengths (500, 1000, 2000 ms) and relative biomarkers values.

Figure S2. Force-frequency dependence of the ToR-ORd+Land model in control conditions. AP (A), CaT (B), and active tension (C) waveforms and relative biomarkers (time to peak (D), time to 90% repolarisation/decay (E), and normalised peak value (F)) are reported for 0.5, 1, and 2 Hz pacing.

Figure S2 shows that, for increasing pacing frequencies, the time to peak of AP, CaT, and active tension slightly declines (Figure S2-D) while the time to 90% repolarisation/decay is significantly shorter (Figure S2-E). Peak values are larger for CaT and active tension, whereas AP peak is slightly lower at higher pacing frequencies (Figure S2-F). This is in agreement with experimental data of AP duration (APD) rate dependence, peak intracellular calcium, and CaT decay constant reported in O’Hara et al. (2011) and Schmidt et al. (1998), as well as active tension data (Mulieri et al., 1992) for steady-state isometric twitch tension and isometric twitch timing parameters.

The calibrated electro-mechanical models retain also good agreement with experimental data in terms of length-dependence properties (Figure S3, Table S4), despite direct comparison with experimental evidence being challenging due to differences between experimental and simulation setups. Vahl et al. (1997) reported an increase in developed force of a factor 5.5 in normal donor hearts going from a of 0.8×Lmax to Lmax. At Lmax the time to peak was 175±23 ms and the time required from peak force to 90% relaxation was 240±25 ms.

Holubarsch et al (1998) showed that, when muscle length was increased from 90% to 100% optimum length, peak developed force increased by 36% in non-failing human left ventricular myocardium.

Figure S3. Length-dependence of action potential (A), calcium transient (B), and active tension (C) simulated using the ToR-ORd+Land model.

Table S4. Quantification of calcium transient and active tension length-dependence properties from simulations using the ToR-ORd+Land model.

|  | **Ca ampl (nM)** | **Ca Tpeak (ms)** | **Ca 90% relax (ms)** | **Ta ampl (kPa)** | **Ta Tpeak (ms)** | **Ta 90% relax (ms)** |
| --- | --- | --- | --- | --- | --- | --- |
| $\boldsymbol{\lambda}$ **= 0.9** | 489.41 | 37.36 | 269.76 | 17.64 | 151.42 | 207.92 |
| $\boldsymbol{\lambda}$ **= 0.95** | 447.19 | 40.02 | 286.69 | 20.44 | 159.42 | 211.53 |
| $\boldsymbol{\lambda}$ **= 1** | 402.86 | 42.3 | 314.84 | 24.0 | 170.43 | 226.21 |
| $\boldsymbol{\lambda}$ **= 1.05** | 356.11 | 46.01 | 351.33 | 28.62 | 180.94 | 250.81 |
| $\boldsymbol{\lambda}$ **= 1.1** | 307.62 | 50.78 | 396.19 | 34.94 | 196.74 | 284.74 |

## **S7. Electro-mechanical window in electro-mechanical and electrophysiology-only models**

The electro-mechanical window (EMw) is defined as the difference between the duration of electrical and mechanical systole. Using electrophysiology-only simulations, it has been previously computed as the difference between the APD and CaT duration at 90% repolarisation/decay (Passini et al., 2019). In our study, we also computed it with the electro-mechanical models using the difference between APD and active tension duration. The results are shown in Figure S4.

Our simulations show that, in baseline conditions, the EMw is shorter in electro-mechanical models than electrophysiology-only models. Moreover, the EMw shortening under Dofetilde exposure is larger for the electrophysiology-only models when computed with calcium, whereas it is larger for the electro-mechanical models when computed with tension. As shown in Figure S4-B, mild APD prolongations lead to increased intracellular calcium amplitudes whereas severe APD prolongation cause abnormalities and decrease in intracellular calcium amplitude. This two-phase effect of Dofetilide on calcium amplitude is mirrored in active tension amplitude. The effect of Dofetilide on calcium amplitude is followed by a steady increase in calcium duration, whereas tension duration increases less then calcium duration under drug exposure and reaches a plateau when drug dose increases. This is particularly important when evaluating the EMw. For the purposes of our study, we compared the EMw of electro-mechanical models against electrophysiology-only models, and to be consistent in the comparison, we adopted the same EMw formulation for both models’ categories (i.e. EMw computed with calcium). This supported our claim on the robustness of repolarisation in electro-mechanical models.

Figure S4. Electro-mechanical window (EMw) in electro-mechanical and electrophysiology-only models. A: The EMw, computed considering calcium or tension, is shorter in electro-mechanical models. The EMw shortening under drug exposure (inset), known to be an effective biomarker of TdP prediction, is larger for the electrophysiology-only models when computed with calcium, whereas it is larger for the electro-mechanical models when computed with tension. B: Drug-induced effects on CaT and active tension waveforms and time to peak and to 90% decay biomarkers.

## **S8. Contractility escapes simulated with the ToR-ORd+Land in tissue**

The ToR-ORd+Land model, as the ORd+Land, demonstrated contractility escapes within the tested range of Dofetilide concentrations (Figure S5).

Figure S5. 3D tissue simulations using the ToR-ORd+Land model at 2 Hz pacing. 0.1 µM of Dofetilide induces APD prolongation (A) that trigger contractility escapes (B). AP and z displacements recorded at the free surface opposite stimulation.

# References

- Dutta, S., Mincholé, A., Zacur, E., Quinn, T.A., Taggart, P., Rodriguez, B., 2016. Early afterdepolarizations promote transmural reentry in ischemic human ventricles with reduced repolarization reserve. Progress in Biophysics and Molecular Biology 120, 236–248. <https://doi.org/10.1016/j.pbiomolbio.2016.01.008>
- Haynes, P., Nava, K.E., Lawson, B.A., Chung, C.S., Mitov, M.I., Campbell, S.G., Stromberg, A.J., Sadayappan, S., Bonnell, M.R., Hoopes, C.W., Campbell, K.S., 2014. Transmural heterogeneity of cellular level power output is reduced in human heart failure. Journal of Molecular and Cellular Cardiology 72, 1–8. <https://doi.org/10.1016/j.yjmcc.2014.02.008>
- Holubarsch, C., 1998. Shortening versus isometric contractions in isolated human failing and non-failing left ventricular myocardium: dependency of external work and force on muscle length, heart rate and inotropic stimulation. Cardiovascular Research 37, 46–57. <https://doi.org/10.1016/S0008-6363(97)00215-0>
- King, N.M.P., Methawasin, M., Nedrud, J., Harrell, N., Chung, C.S., Helmes, M., Granzier, H., 2011. Mouse intact cardiac myocyte mechanics: cross-bridge and titin-based stress in unactivated cells. Journal of General Physiology 137, 81–91. <https://doi.org/10.1085/jgp.201010499>
- Land, S., Park-Holohan, S.-J., Smith, N.P., dos Remedios, C.G., Kentish, J.C., Niederer, S.A., 2017. A model of cardiac contraction based on novel measurements of tension development in human cardiomyocytes. Journal of Molecular and Cellular Cardiology 106, 68–83. <https://doi.org/10.1016/j.yjmcc.2017.03.008>
- Mulieri, L.A., Hasenfuss, G., Leavitt, B., Allen, P.D., Alpert, N.R., 1992. Altered myocardial force-frequency relation in human heart failure. Circulation 85, 1743–1750. <https://doi.org/10.1161/01.CIR.85.5.1743>
- O’Hara, T., Virág, L., Varró, A., Rudy, Y., 2011. Simulation of the Undiseased Human Cardiac Ventricular Action Potential: Model Formulation and Experimental Validation. PLoS Computational Biology 7, e1002061. <https://doi.org/10.1371/journal.pcbi.1002061>
- Passini, E., Trovato, C., Morissette, P., Sannajust, F., Bueno‐Orovio, A., Rodriguez, B., 2019. Drug‐induced Shortening of the Electromechanical Window is an Effective Biomarker for *in Silico* Prediction of Clinical Risk of Arrhythmias. British Journal of Pharmacology 176, 3819–3833. <https://doi.org/10.1111/bph.14786>
- Schmidt, U., Hajjar, R.J., Helm, P.A., Kim, C.S., Doye, A.A., et al., 1998. Contribution of abnormal sarcoplasmic reticulum ATPase activity to systolic and diastolic dysfunction in human heart failure. Journal of Molecular and Cellular Cardiology 30: 1929–1937.
- Tomek, J., Bueno-Orovio, A., Passini, E., Zhou, X., Minchole, A., Britton, O., Bartolucci, C., Severi, S., Shrier, A., Virag, L., Varro, A., Rodriguez, B., 2019. Development, calibration, and validation of a novel human ventricular myocyte model in health, disease, and drug block. eLife 8, e48890. <https://doi.org/10.7554/eLife.48890>
- Vahl, C.F., Timek, T., Bonz, A., Kochsiek, N., Fuchs, H., Sch�ffer, L., Rosenberg, M., Dillmann, R., Hagl, S., 1997. Myocardial length-force relationship in end stage dilated cardiomyopathy and normal human myocardium: analysis of intact and skinned left ventricular trabeculae obtained during 11 heart transplantations. Basic Research in Cardiology 92, 261–270. <https://doi.org/10.1007/BF00788521>
